# Allelic exclusion of the immunoglobulin heavy chain locus is independent of its nuclear localization in mature B cells

**DOI:** 10.1093/nar/gkt491

**Published:** 2013-06-07

**Authors:** Sjoerd J. B. Holwerda, Harmen J. G. van de Werken, Claudia Ribeiro de Almeida, Ingrid M. Bergen, Marjolein J. W. de Bruijn, Marjon J. A. M. Verstegen, Marieke Simonis, Erik Splinter, Patrick J. Wijchers, Rudi W. Hendriks, Wouter de Laat

**Affiliations:** ^1^Hubrecht Institute-KNAW & University Medical Center Utrecht, Utrecht 3584 CT, The Netherlands and ^2^Department of Pulmonary Medicine, Erasmus MC Rotterdam, Rotterdam, Box 2040, 3000 CA, The Netherlands

## Abstract

In developing B cells, the immunoglobulin heavy chain (*IgH*) locus is thought to move from repressive to permissive chromatin compartments to facilitate its scheduled rearrangement. In mature B cells, maintenance of allelic exclusion has been proposed to involve recruitment of the non-productive *IgH* allele to pericentromeric heterochromatin. Here, we used an allele-specific chromosome conformation capture combined with sequencing (4C-seq) approach to unambigously follow the individual *IgH* alleles in mature B lymphocytes. Despite their physical and functional difference, productive and non-productive *IgH* alleles in B cells and unrearranged *IgH* alleles in T cells share many chromosomal contacts and largely reside in active chromatin. In brain, however, the locus resides in a different repressive environment. We conclude that *IgH* adopts a lymphoid-specific nuclear location that is, however, unrelated to maintenance of allelic exclusion. We additionally find that in mature B cells—but not in T cells—the distal V_H_ regions of both *IgH* alleles position themselves away from active chromatin. This, we speculate, may help to restrict enhancer activity to the productively rearranged V_H_ promoter element.

## INTRODUCTION

B and T lymphocytes express a large repertoire of antigen receptors that safeguard the robustness of our adaptive immune response. Lymphocyte development uniquely relies on scheduled genomic rearrangement of V (variable), D (diversity) and J (joining) gene segments in the antigen receptor loci (1–3).

The murine *IgH* locus spans nearly ∼3 Mb, with upstream ∼150 functional V_H_ segments spread over ∼2.4 Mb, followed by D_H_ and J_H_ segments and a ∼200 kb constant (C_H_) gene region. V(D)J recombination, initiated by the recombination activating gene-1 (Rag1) and Rag2 proteins, is regulated at three different levels: (i) cell lineage-specificity, (ii) temporal order within a lineage and (iii) allelic exclusion, which is the mechanism that guarantees that only one receptor is expressed per lymphocyte (2–4). The *IgH* locus contains many *cis*-regulatory elements, including the intergenic control region 1 (IGCR1), the intronic enhancer E_µ_ and the downstream 3′ regulatory region (3′RR), which are involved in the regulation of the of V(D)J recombination (5–7) and class switch recombination (8). Chromosome topology and nuclear location have been implicated in the control of V(D)J recombination and allelic exclusion (3,9–11). In the early pro-B stage, the *IgH* locus adopts a central position in the nuclear interior and chromatin looping mediates physical proximity of both ends of the locus (12,13), facilitating recombination of distal V_H_ genes (13–16). Succesfull D_H_-to-J_H_ recombination on both alleles is followed by productive V_H_ to D_H_J_H_ recombination on only one allele. Prohibition of further rearrangement of the other allele, called allelic exclusion, is thought to be controlled by multiple (partly) redundant and successive mechanisms (17). In pre-B cells, on successful V(D)J rearrangement both *IgH* loci decontract and the non-productive allele is seen to relocate to pericentromeric heterochromatin (PCH) (15). No heterochromatin tethering was observed in early pro-B cells prior to rearrangement, nor in resting splenic B cells, suggesting that mono-allelic recruitment to heterochromatin is developmentally controlled (18). Only on activation of splenic B cells, mono-allelic *IgH* recruitment to PCH appears to re-occur (18). Mono-allelic expression was reported to take place preferentially from the non-associated allele, suggesting that recruitment to heterochromatin helps to maintain silencing of the non-productive *IgH* allele (18). In contrast with these findings, it has also been reported that activated splenic B cells transcribe both *IgH* alleles (19). To what extent the two *IgH* alleles in mature B cells differ therefore remains unclear.

While FISH enables studying locus positioning at the single cell level, it is limited in throughput and provides relatively low resolution spatial information. Chromosome conformation capture (3C) technology (20) has been applied to study *IgH* locus conformation in more detail. 3C revealed two major contacts in the unrearranged *IgH* locus, one between E_µ_ and 3′RR, and the other between E_µ_ and IGCR1 (5,21). The CCCTC-binding factor CTCF (22) and cohesin were implicated in these loops, which appear to create a topological subdomain that covers the region from 3′RR to IGCR1 (5,21). The proximal and distal V_H_ region also adopt distinct topological substructures that then merge with the 3′ domain to maximize D_H_J_H_ contacts with the full V_H_ gene repertoire (16,23). Thus, in early B cell development, *IgH* topology ensures that proximal and distal V_H_ genes have equal opportunites to interact with E_µ_. In mature B cells that have completed V(D)J recombination, however, the chromatin structure of *IgH* is expected to be different, as promiscuous interactions of E_µ_ with numerous upstream V_H_ promoters may interefere with accurate and efficient transcription from the functionally rearranged V_H_ promoter.

In this study, we characterized the structural properties and genomic environments of the productive and non-productive *IgH* allele separately. We applied allele-specific 4C-seq (24,25) to compare at high resolution the chromatin configuration of the productive and non-productive *IgH* alleles in mature B cells, as well as the unrearranged *IgH* alleles in T cells and non-lymphoid cells. We also evaluated *IgH* nuclear positioning, as determined by the genomic contacts formed by these alleles.

## MATERIALS AND METHODS

### Separation and stimulation of IgM^a^- and IgM^b^-expressing B cells

*Magnetic activated cells sorting (MACS)*: mature resting B cells were purified using streptavidin-coupled magnetic beads (Miltenyi Biotec), by negative selection using the following biotinylated antibodies: CD5, CD43, CD138, CD11b, Gr-1 and TER-119 (Supplementary Table S1.1).

*Fluorescence activated cells sorting (FACS)*: Separation of B cell populations based on IgM allotype expression was done by FACS sorting of MACS-purified fractions of resting B cells using antibodies to B220 and CD19, in conjunction with allotype-specific antibodies for IgM^a^ [FVB] and IgM^b^ [C57BL/6], for details see Supplementary Table S1.3.

*In-vitro activation*: Purified (resting) B cells were *in-vitro* activated, as described (18) for 4 days using αCD40-coated plates (20 µg/ml; BD Biosciences) and IL-4 (IL-4 50 ng/ml; Peprotech). For further details see Supplemental Data.

### 4C template preparation & mapping

FACS-sorted cells were used for 4C template preparation. Cells were fixed and lysed as described (26) using HindIII (Roche) as a first cutter and DpnII (New England Biolabs) as a second cutter. An allele-specific strategy for single-end 4C-sequencing was used as described (25,27), where restriction fragment length polymorphisms between the C57Bl/6 and the FVB genome are exploited. Primers for the single-end 4C-seq experiments were designed around an SNP that creates an extra DpnII restriction enzyme site on the FVB allele. Consequently, only the C57Bl/6 allele will be analyzed using this strategy (Supplementary Figure S2). For the allele-specific paired-end 4C sequencing (PE-4Cseq), primers were designed such that one of the selected primers read an SNP (P2) (28) whereas the other primer (P1) read into the captured sequence ligated to the ‘bait’ fragment in the 4C procedure. Consequently, simultaneous analysis of two alleles is possible. The single-end data were mapped, allowing no mismatches, to a database of 4C-seq fragment ends generated from the mm9/NCBI m37 version of the mouse genome (29). The paired-end sequencing data were first split based on the SNPs (C57Bl/6 vs FVB) detected in the second read (PE2) of the read-pair and subsequently the first read of the pair (PE1) was mapped as single-end data. Significant genomic contacts, visualized by domainograms, were identified based on described algorithms (29). For further details see Supplementary Data.

### RNA-FISH and DNA-FISH

MACS-sorted cells were used for RNA and DNA FISH experiments. RNA and DNA FISH experiments were performed as described (27), with minor adjustments. Briefly, for DNA FISH, denaturing of the DNA in the cells on slides was done for 10’ @ 80°C in 50% formamide / 2× SSC after which a denatured probe was applied to the slide for overnight hybridization at 42°C followed by post hybridization washes and microscopic analysis. For further details see Supplemental Data.

For detailed description of mice, B and T cell isolation, lymphoid cell culture, separation of IgM^a^- and IgM^b^-expressing B cells, 4C-seq procedures, RNA expression analysis, RNA-FISH and DNA-FISH, see Supplementary Materials and Methods.

## RESULTS

### Both *IgH* alleles are transcribed and positioned similarly in resting and activated splenic B cells

To obtain a pure B cell population from spleen, we used the cell-sorting strategy described in (18) but included additional markers to further exclude non-B cells (see materials and methods). Because recruitment of non-productive *IgH* alleles to PCH was observed in activated but not in resting B cells (18), we performed 4C-Seq experiments both in resting and αCD40-activated splenic B cells. Proper activation of splenic B cells was verified by gene profiling, demonstrating upregulation of key genes including *Aicda*, *IL-5R*, *CD44*, *Fas*, *c-Myc* and *cyclin D2* (Supplementary Table S2).

**Figure 1.**
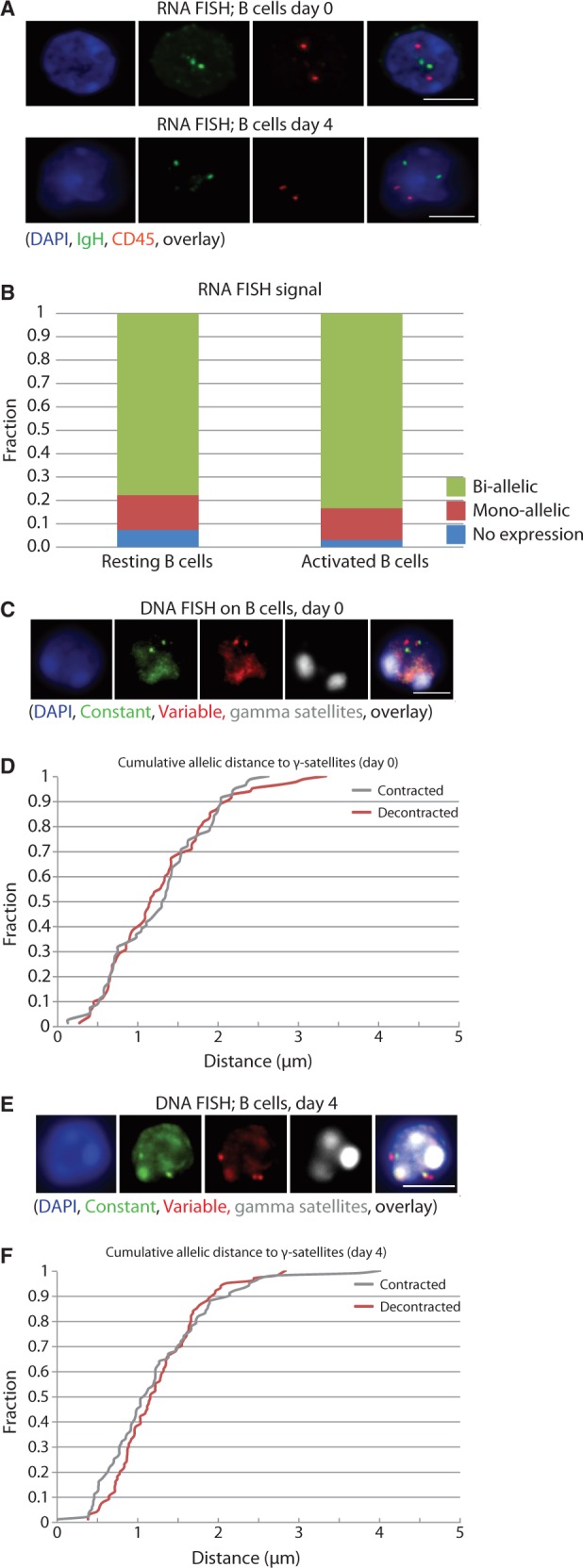
Biallelic expression and comparable nuclear positioning of the two *IgH* loci relative to PCH in resting and activated B cells. (**A**) Representative picture of RNA FISH in resting (*upper row*) and activated B cells (*lower row*). (**B**) Quantification of the RNA FISH data plotted as the percentage of *IgH* signals in CD45^+^ cells on the Y-axis. A minimum of 50 cells were analyzed per cell type. Cells with >2 RNA signals for *IgH* were excluded from the analysis. (**C**) Representative picture of DNA FISH in resting B cells. (**D**) Cumulative frequency of the minimal distance of *IgH* signals to the γ-satelite FISH signal in resting B cells. Contraction is defined as the minimal distance between two different probes on the *IgH* locus. The distance of the *IgH* signals to γ-satelites in μm is depicted on the X-axis. (**E**) Representative picture of DNA FISH in activated B cells. (**F**) Cumulative frequency of the minimal distance of the *IgH* signals to the γ-satelite FISH signal in activated B cells. FISH pictures represent several Z-stacks projected on top of each other, scale bar in overlays depicts 4 µm. Images were collected using a Leica DM6000 B microscope equipped with a 100× objective, Leica DFC360 FX camera, taking z-steps of 0.2 µm. Leica application suite 2.6.0 software was used both for image collection and deconvolution.

First, we tested *IgH* expression by RNA-FISH with a BAC probe spanning the Cµ–J_H_–D region. Both in resting and in activated B cells, we detected biallelic expression in ∼75–80% of cells (Figure 1A and B). RNase-treated cells showed no signals, demonstrating that we were measuring RNA (Supplementary Figure S1). These results supported previously published work in which biallelic expression (19,30,31), active chromatin marks and RNA polymerase II binding (30) were found to be associated with both productive and non-productive *IgH* alleles in mature B cells.

We then asked whether B cell stimulation is accompanied by allele-specific repositioning of the non-productive *IgH* locus to PCH, as previously reported (18). We performed DNA FISH and measured *IgH* distances relative to PCH, as stained for with a γ-satellite probe. To discriminate productive from non-productive alleles, we visualized both *IgH* ends and assumed that the most contracted locus represented the productive allele (15). In resting B cells, none of the *IgH* alleles showed striking PCH proximity (Figure 1C and D). In stimulated B cells, the non-productive locus appeared a bit more frequently near PCH than the productive allele, but physical contacts within the 300 nm range were rare for both alleles (Figure 1E and F).

These microscopy studies therefore indicate that in resting and activated splenic B cells both *IgH* loci are transcribed and that none of the two *IgH* loci are closely associated with PCH.

### Allele-specific 4C-seq strategy to analyze productive and non-productive *IgH* alleles

**Figure 2.**
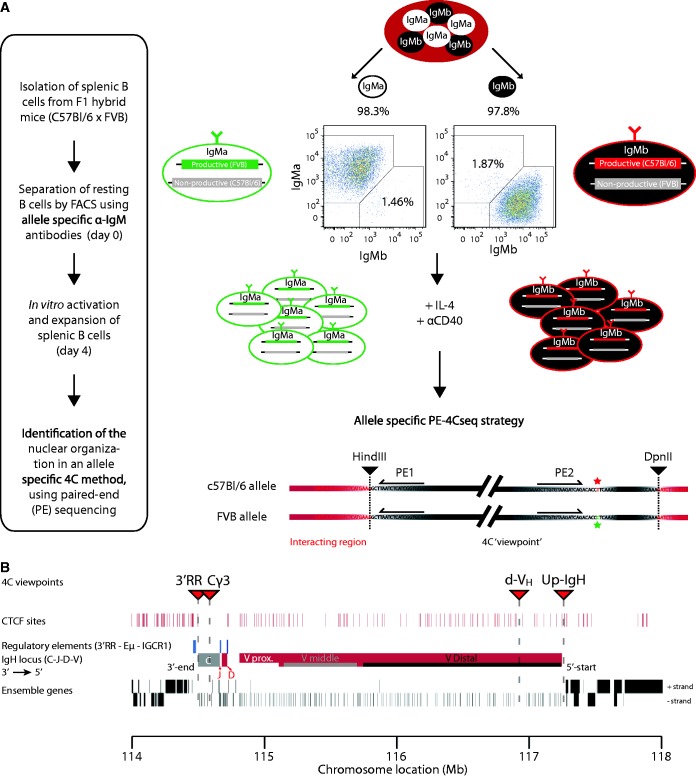
Allele-specific 4C-seq strategy allowing separate analysis of productive and non-productive *IgH* alleles in B cells. (**A**) Schematic view of the experimental approach. The purity of the IgM^a^/IgM^b^-allotype-sorted populations is depicted above the FACS plots. The allele-specific 4C-seq strategy shows a schematic 4C ‘viewpoint’. The restriction sites are depicted in black triangles; the red and green stars highlight the SNP between the C57BL/6 and the FVB allele, respectively. The 4C viewpoint (black) with its captured interactions (red) is not drawn to scale. (**B**) Schematic view of the *IgH* locus highlighting the 4C viewpoints (red triangles), CTCF sites in mature B cells (GEO accession: GSM672402), regulatory elements (blue bars), the C region (grey), the J_H_, D and V_H_ regions (red), and the Ensemble genes (black bars) on mouse chromosome 12 (mm9). The proximal (Vprox), middle (Vmiddle) and distal (Vdistal) region of the variable region are indicated with white, grey and black bars, respectively. The locus is drawn to scale. Mb = Megabase.

To identify cells exclusively expressing the paternally or maternally derived *IgH* allele, we took advantage of IgM allotype differences: B cells from FVB or C57BL/6 mice produce heavy chains of the IgM^a^ or IgM^b^ allotype, respectively, which differ in a single amino acid (32). We used allotype-specific antibodies in FACS to sort separate pools of IgM^a^- and IgM^b^-expressing splenic B cells from (FVB[IgM^a^] × C57BL/6[IgM^b^]) F1 mice. Both resting and αCD40-activated splenic B cell fractions were sorted into two populations: one with cells carrying a productive FVB[IgM^a^] and a non-productive C57BL/6 allele, and another with cells carrying a productive C57BL/6[IgM^b^] and a non-productive FVB allele (Figure 2A).

To independently analyze the topology of these two functionally and physically different *IgH* alleles, we used allele-specific 4C-seq technology. 4C-seq enables the generation of genome-wide DNA contact profiles of a chromosomal sequence of interest, called the ‘viewpoint’ (24,25). In this allele-specific 4C-seq variant, we took advantage of C57BL/6 or FVB haplotype-specific single nucleotide polymorphisms (SNPs). We designed a strategy based on paired-end (PE) sequencing, whereby PE1 analyzes 4C ligation products and therefore identifies DNA contact partners, while PE2 reads a SNP inside the ‘viewpoint’ fragment and therefore links the PE1 contact profile to either the C57BL/6 or the FVB allele. Thus, the paired-end 4Cseq strategy (PE-4Cseq) enables independent but simultaneous analysis of both alleles, which is different from a previously developed method that uses single-end 4Cseq (SE-4Cseq) to analyze only one of the two alleles in a cell population (25). Three PE-4Cseq viewpoints were designed: *3′RR*, near the upstream 3′ regulatory region; *Cγ3*, inside the Cγ3 region and *d-V_H_*, in the distal-V_H_J558 region. A fourth viewpoint, *Upstream-IgH (Up-IgH)*, at the 5′ end of *IgH* just beyond the most distal V_H_ gene (Figure 2B), was used for allele-specific analysis based on SE-4Cseq (Supplementary Figure S2). The B cell populations studied consist of cells with differently rearranged *IgH* alleles. Three of the four 4Cseq viewpoints reside outside the V(D)J rearranged part of *IgH* and therefore enable DNA contact assessment of both alleles independent of their rearrangement. Only the *d-V_H_* viewpoint resides just inside the distal-V_H_J558 region and may therefore miss a few rearranged alleles.

### Topology of the *IgH* locus

We generated DNA contact profiles in resting and αCD40-activated splenic B cells, resting and αCD3-activated splenic T cells as well as fetal brain cells (serving as a non-lymphoid control). All 4C-seq profiles showed the typical contact distribution expected from polymer physics, with high contact frequencies between sequences close on the linear chromosome and with intrachromosomal captures being preferred over interchromosomal contacts (Supplementary Figure S3) (29). C57BL/6- and FVB-specific 4C-seq profiles were essentially identical; we show C57BL/6-specific profiles, unless specified as in Supplementary Figure S4.

**Figure 3.**
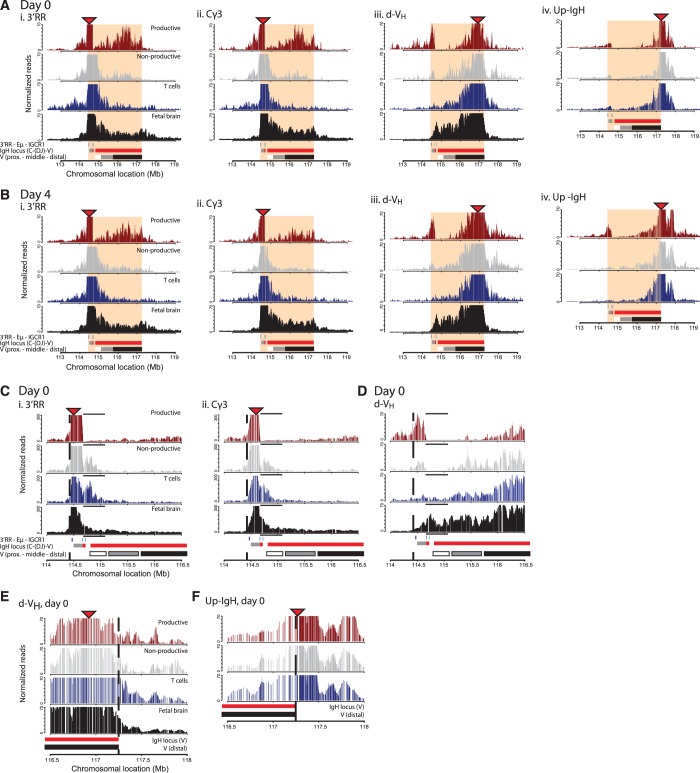
4C-seq profiles reveal allele-specific contacts in B cells and high-resolution definition of the topological domain spanning the *IgH* locus. (**A**) Contact profiles in resting B cells for the C57BL/6 allele across the *IgH* locus looking from four different 4C viewpoints depicted by red triangles, from left to right: *3′RR* (i), *Cγ3* (ii), *d-V_H_* (iii) and *Up-IgH* (iv). Per viewpoint the different tissues are plotted (*from top to bottom*): B cell productive allele (red) and non-productive allele (grey), T cell (blue) and fetal brain (black). A schematic representation showing the regulatory elements (blue), the C (grey) and V(red) regions of the IgH locus and the different regions within the V-region (proximal—middle—distal), as well as the chromosomal location in Megabase (Mb) is given at the bottom. The topological domain is depicted in shaded red. The Y-axis represents the normalized captured sequencing reads analyzed with a running median of 21 HindIII fragments, in arbitrary units. (**B**) Contact profiles in activated B cells. (**C**) Zoom of the 3′-border (*dotted line*) of the topological domain looking from the *d-V_H_* 4C viewpoint in resting splenic B cells. The black line above each track indicates the region where the productive allele in B cells has only few interactions compared with the other alleles. (**D**) Zooms of the 3′-border (*dotted line)* of the topological domain looking from the *3′RR* (i) and the *Cγ3* (ii) 4C viewpoints (*red triangles*). (**E**) Zoom of the 5′-border (*dotted line*) of the topological domain looking from the *d-V_H_* (i) and the (**F**) *Up-IgH* 4C viewpoints (*red triangles*) in resting splenic B cells.

In all cell types and for all three internal *IgH* viewpoints, the most abundant local contacts appeared confined to the ∼3 Mb *IgH* locus (Figure 3A and B). In B lymphocytes, contacts made between the two ends of the locus were particularly frequent (Figure 3D), which probably reflects close linear proximity as a consequence of V_H_-to-DJ_H_ recombination. Rapid drops in contact frequencies suggestive of structural boundaries were seen at either end of the locus. The 3′ *IgH* boundary is best appreciated by the 4C-seq plots of the two closest viewpoints, *3′RR* and *Cγ3*. Both showed loss in contact frequencies just beyond the 3′RR (Figure 3A–C). The 5′ *IgH* boundary is evident from the contact profiles of the two 5′ viewpoints. *d-V_H_*, inside the *IgH* locus, preferentially captured *IgH* sequences but shows a clear reduction in contacts just beyond the last *V_H_* gene (Figure 3A–E). By contrast, *Up-IgH*, just outside the *IgH* locus, showed a strong preference to capture sequences further away from *IgH* and did not make frequent contacts within *IgH *(Figure 3A, B and F)*.* These findings suggested that the *IgH* locus forms a spatially distinct entity in mature B cells, as was described for pro-B cells (16), that is similar to the previously described topological domains identified by Hi-C (33–36) (Supplementary Figure S5). This structural organization was identified in cycling and non-cycling B and T lymphocytes, as well as in fetal brain cells.

### Recombination and chromatin looping in the *IgH* domain

Further inspection of 4C-seq profiles revealed additional tissue-specific and allele-specific structural features of the *IgH* locus. The most distinct conformation was adopted by the productive allele in B cells, whereby the 3′ viewpoints showed a complex landscape of frequent contacts across the middle and distant V regions (Figure 3A and B). Strong peaks in this landscape did not appear to cover specific locations, e.g. they did not coincide with the regulatory Pax5-binding PAIR elements (37). The *3′RR* and *Cγ3* viewpoints showed few interactions with the ∼0.3 Mb region containing the D segments, the IGCR1 element and proximal V_H_ genes (Figure 3A–C). Also, when looked from *d-V_H_*, extensive loss of 4C signals in resting and stimulated B cells was seen across a large region containing the proximal V_H_ genes (Figure 3A–3D). Signals were strongly reduced but not completely absent (see Supplementary Figure S6 for underlying raw 4C-seq data). When looked from the same distal viewpoint (Figure 3C), reduced signal across the proximal V region was also seen at the non-productive IgH allele. However, 3′RR and *Cγ3* show frequent interactions with non-rearranged sequences in this proximal ∼0.3 Mb region (Figure 3C). These findings would be in agreement with frequenct DJ_H_ configurations on the non-productive allele [present in ∼50% of B cells (38–41)], as well as frequent rearrangement to the proximal V_H_7183 family. The latter is conceivable, as it is known that V_H_7183-family genes are preferentially rearranged, but selected against cellularly because of their incompetence to form a pre-BCR (42,43). Interestingly, the *IgH* locus in T cells structurally best resembles the non-productive *IgH* allele in B cells, indicating proximity of the 3′RR and Eµ regions and the proximal V_H_ genes in T cells (Figure 3A–D). Collectively, these results validate that our approach truly analyzes the productive and non-productive allele separately, show that the locus forms a large topological domain in all cell types analyzed, and confirm that large-scale chromosomal rearrangements take place specifically at the productive allele.

### The *IgH* locus is in a similar chromatin compartment in B and T lymphocytes

Microscopy studies have suggested that the *IgH* locus switches between positions inside the cell nucleus, involving recruitment to nuclear periphery or PCH, in a cell-type and allele-specific manner (12,18). We reasoned that such different locations should result in different chromatin environments, which can be assessed based on the long-range intra- and interchromosomal contacts measured by 4C-seq.

**Figure 4.**
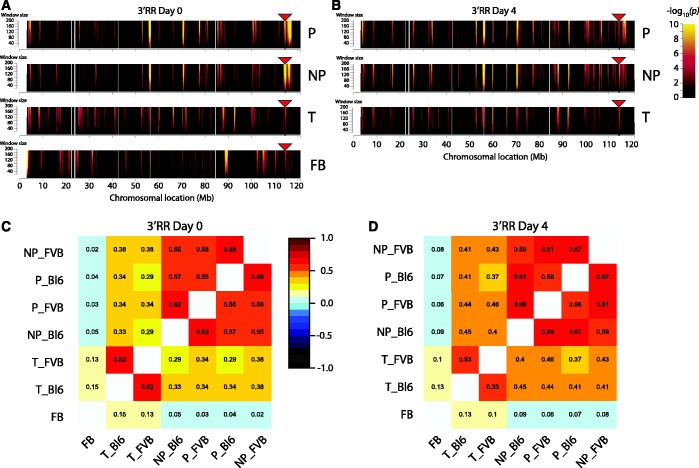
The *IgH* locus shows similar long-range contacts along chromosome 12 between the different alleles in lymphocytes. (**A**) Domainograms showing chromosome-wide interaction profiles looking from the *3′RR* 4C viewpoint (*red triangle*) on the *IgH* locus in resting B cells, from top to bottom: B cell productive (P), B cell non-productive (NP), T cell (T) and fetal brain allele (FB). Significance of the interactions is indicated by the range in colour used in the domainogram as depicted in the legend: black is low significance (*P* = 1) and yellow represents high significance (*P* = 10^-10^) of interaction. Window size of the running window analysis is depicted on the Y-axis. (**B**) Chromosome-wide interaction profiles looking from the *3′RR* 4C viewpoint (*red triangle*) in activated B cells. (**C**) Correlation plot of the interactions looking from the *3′RR* 4C viewpoint in resting B cells (window size 21). The numbers represent the Spearman rank correlation coefficient, colours range from linear anti-correlation (black) to linear correlation (dark red). Tissues and separate alleles are coded as follows: B cell non-productive (NP), B cell productive (P), T cell (T), fetal brain (FB), C57BL/6 allele (Bl6) and FVB allele (FVB). (**D**) Correlation plot of the interactions looking from the *3′RR* 4C viewpoint in activated B cells (window size 21).

Chromosome-wide contact profiles revealed preferred contacts with specific regions across chromosome 12 (the chromosome that contains *IgH*), both in resting and stimulated B lymphocytes (shown for *3′RR* in Figure 4A and B, respectively; for FVB profiles see Supplementary Figure S7). DNA-FISH was performed to validate these results (SupplementaryFigure S8). Correlation plots of the 4C results show that the productive and non-productive *IgH* alleles in B cells were engaged in similar intra-chromosomal contacts, as apparent from all viewpoints (Figure 4C and D; Supplementary Figures S9 and 10) and that many of these regions were also contacted in T cells. This is surprising, as the locus is thought to be differentially positioned in B and non-B cells (12). By contrast, in a non-lymphoid tissue, fetal brain, the *IgH* locus clearly made different contacts that even appeared mutually exclusive between brain and lymphocytes (Figure 4A). Not only intra-chromosomal, but also inter-chromosomal contacts corresponded between the productive and non-productive allele in B cells and those made by *IgH* in T cells, while the locus formed entirely distinct *trans*-contacts in fetal brain (Supplementary Figure S11).

Taken together, these data suggest that irrespective of its transcriptional or recombinational state, *IgH* is positioned in a similar chromatin compartment in splenic B and T lymphocytes, which is different from its chromatin environment in brain tissue.

### The 5′ and 3′ end of the *IgH* locus are in different chromatin environments

**Figure 5.**
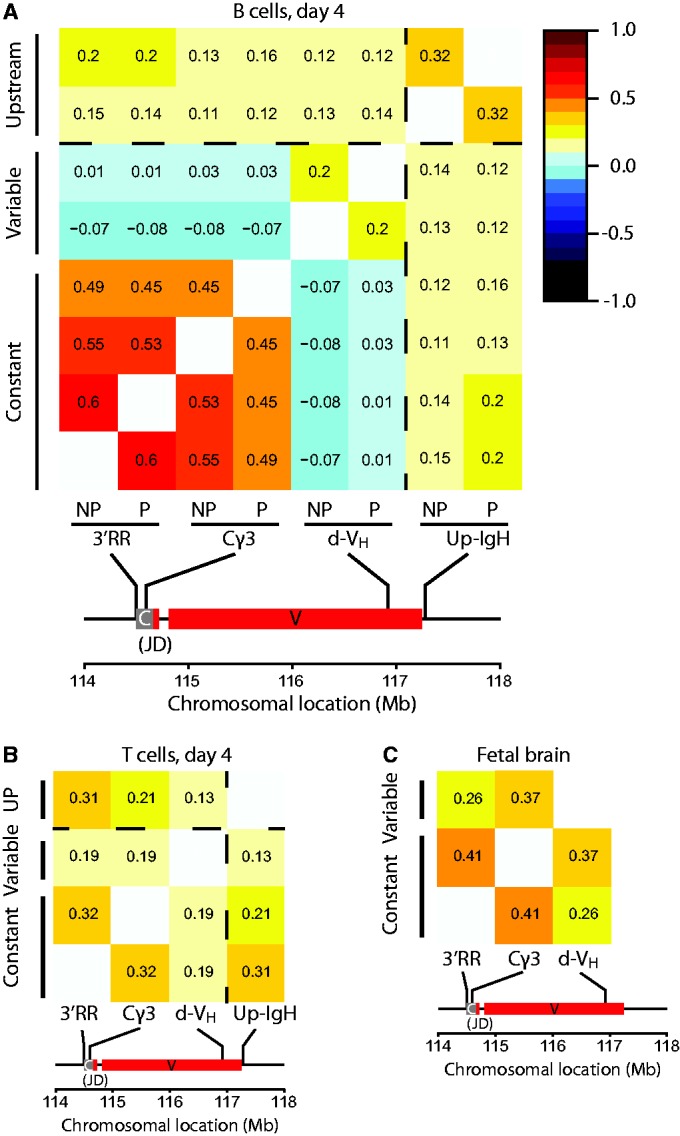
Genome-wide contacts are not uniform across different regions of the *IgH* locus in B cells. (**A**) Correlation plot of the interactions of C57BL/6 viewpoints in *cis*, in activated B cells. The X-axis shows the productive (P) and the non-productive (NP) C57Bl/6 alleles looking from the four 4C viewpoints. The *IgH* locus with the position of the 4C viewpoints is drawn to scale below the X-axis. The numbers represent the Spearman rank correlation coefficients, colours range from linear anti-correlation (black) to linear correlation (dark red). The dotted line represents the topological border that is present between the *d-V_H_* and the *Up-IgH* 4C viewpoints. Correlation values are given between 1 and −1. A correlation of 1 means perfect correlation, a correlation of 0 means uncorrelated, and a correlation of −1 means perfect anti-correlation. (**B**) Correlation plot of the interactions of C57BL/6 viewpoints in *cis* in resting T cells. (**C**) Correlation plot of the interactions made in fetal brain cells.

Reported 3D FISH analyses indicated that—when recruited to heterochromatin—the *IgH* locus was oriented in such a way that the distal V_H_J558 gene family was positioned closer to the γ-satellite cluster than the proximal V_H_7183 or Cγ1 genes (15). It is therefore conceivable that 5′ and 3′ ends of *IgH* show different chromosome-wide contact profiles. Correlation plots of the total interactions in *cis* between the four individual viewpoints indeed demonstrated that in B lymphocytes chromosomal contacts formed by the 3′ end of *IgH* (*3′RR* and C*γ3* viewpoints) were very different from those formed by its 5′ end (the *d-V_H_* viewpoint) (Figure 5A for activated B cells; Supplementary Figure S12 for resting B cells)*.* In these correlation analyses, the productive and non-productive *IgH* allele did not differ. Intriguingly, contacts made by the region just upstream of *IgH* resembled those of the 3′ viewpoints more than those made by its linearly close neighbor viewpoint *d-V_H_* (Figure 5A). By contrast, in both T cells and brain cells, chromosomal contacts formed across the entire *IgH* locus were quite similar, with no exceptional profile seen for the distal V region (Figure 5B and C).

Thus, specifically in B cells the *IgH* locus shows remarkable flexibility with the distal V_H_ regions of both *IgH* alleles being in a chromatin environment that is significantly different from either the 3′end of *IgH* or the upstream *IgH* flanking region.

### The distal V_H_ region of both *IgH* alleles is positioned away from active chromatin in B cells

**Figure 6.**
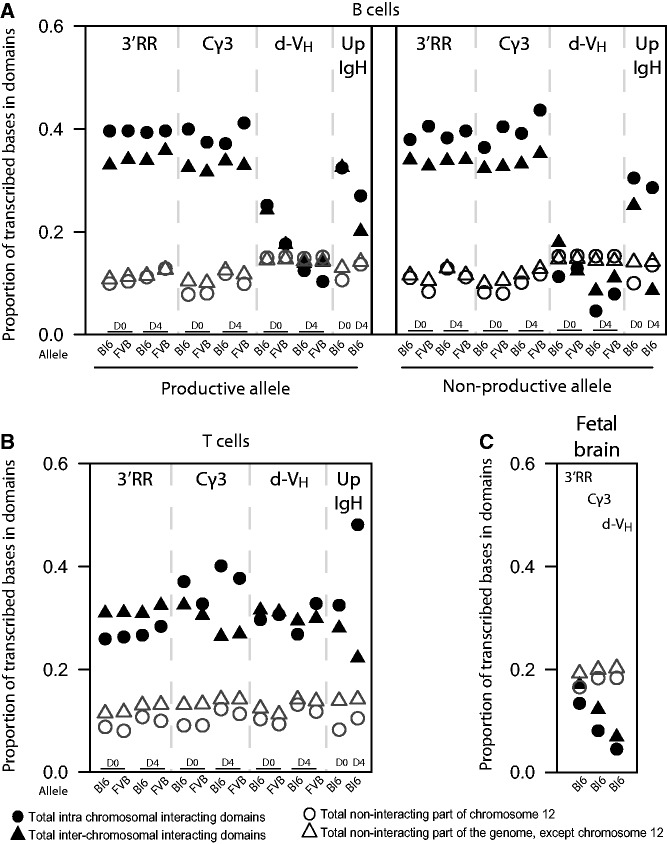
Genome-wide contacts of C_H_ and V_H_ regions in B cells show differences in transcriptional activity. (**A**) Quantification of the transcriptional activity of the interacting domains in *cis* (*filled circles*) and trans (*filled triangles*) contacted by four 4C viewpoints (*indicated at the top*) from the productive allele and non-productive allele in resting and activated B cells. Viewpoints are separated by a dotted line, from left to right: *3′RR*, *Cγ3*, *d-V_H_* and *Up-IgH*. Values for resting cells (D0) and activated cells (D4) are shown per viewpoint. Open circles and open triangles represent the transcriptional activity of the non-interacting regions in *cis* and trans, respectively. The proportion of transcribed bases in the interacting domains is depicted on the Y-axis. (**B**) Quantification of the transcriptional activity of the interacting domains in resting and activated T cells for the four different viewpoints. (**C**) Quantification of the transcriptional activity of the interacting domains in fetal brain for three different viewpoints.

To further characterize the *IgH* chromatin environments, we analyzed the transcriptional activity of regions contacted by *IgH*. We first analyzed the transcriptomes of our resting and stimulated splenic B and T cells in more depth. Hierarchical clustering confirmed the specific expression of B and T cell genes in the corresponding cell types and showed the upregulation of cell-cycle genes after stimulation (Supplementary Figure S13 and Supplementary Tables S3–11). We compared 4C-seq data with matched transcriptome data to analyze the transcriptional activity in contacted regions. In resting and stimulated splenic B cells, intra- and interchromosomal regions contacted by the 3′ part of *IgH* showed relatively high transcriptional activity, when compared with non-contacted parts of the genome (Figure 6A). Surprisingly, this was true not only for the productive, but also for the non-productive *IgH* allele in B cells, and even for the inactive *IgH* locus in T cells (Figure 6A and B). By contrast, regions contacted by *IgH* in brain were relatively inactive, when compared with the remainder of the genome (Figure 6C). Thus, these data show that the 3′ part of the *IgH* locus switches from an inactive chromatin environment in brain cells to an active compartment in B and T lymphocytes, where it resides irrespective of its recombination and transcriptional status.

Flexibility of the *IgH* locus became further apparent when we analyzed the chromosomal contacts formed by the *d-V_H_* and *Up-IgH* viewpoints. In resting and stimulated T cells, as well as in brain cells, chromosomal contacts were similar in transcriptional activity no matter whether they were assessed from the 3′ or the 5′ side of the locus (Figure 6B and C). These findings indicate that the entire *IgH* locus positions itself as a single entity in an inactive environment in brain cells and in an active compartment in T cells. Surprisingly, this was not the case in B cells: *d-V_H_* did not necessarily contact active chromatin, as it did in T cells, but located to a more ‘neutral’ chromatin environment with chromosomal regions that were not different in transcriptional output from the remainder of the genome. Strikingly, this was only seen for *d-V_H_*, as the *Up-IgH* viewpoint again contacted active chromosomal regions. This specific positioning was observed both for the productive and the non-productive *IgH* locus, and both in resting and stimulated B cells.

Thus, in mature B cells, the two ends of both *IgH* loci position themselves in different chromatin environments, with the distal V_H_ region being positioned away from active chromosomal parts.

## DISCUSSION

The specificity of B cell responses during infection relies on extensive antibody diversity whereby each mature B cells bears a single unique type of B cell receptor. Monospecificity of B lymphocytes is ensured by allelic exclusion during V(D)J recombination events in B cell development, which results in the generation of mature B cells with one productively and one non-productively rearranged *IgH* allele. Different mechanisms are thought to regulate mono-allelic *IgH* expression in mature B cells. In particular, mono-allelic recruitment to PCH was proposed to contribute to the maintenance of silencing of the non-productive *IgH* allele (15,18). On the other hand, it was reported that in proliferating splenic B cells more than half of the *IgH* alleles are located at the nuclear periphery, whereby a ∼1 Mb distal V_H_ region regularly colocalizes with the nuclear lamina (44). In 3D-FISH studies, the *IgH* locus was found to be peripheral also in non-B cells, whereby in EL-4 T cells both *IgH* alleles were associated with the nuclear lamina but not with PCH (12,44). The proposed model of mono-allelic recruitment to PCH in mature B cells (15,18) was further challenged by the observation that both productive and nonproductive *IgH* alleles are transcribed in activated splenic B cells (19,30).

Whereas FISH enables the analysis of locus positioning at the single-cell level, it is limited in throughput and provides relatively low-resolution spatial information. In our study, we used an allele-specific 4C-seq strategy, based on IgM^a^/IgM^b^ allotypes and the C57BL/6- or FVB-specific SNPs they contain. Using this strategy, we analyzed in great detail the genome-wide contacts made by the functionally different *IgH* alleles in splenic B cells, as well as in T cells and non-lymphoid cells. In contrast with published microscopy observations (15,18), we found that (i) the two physically different *IgH* alleles in splenic B cells occupy the same chromatin compartments; (ii) the overall chromatin environment of *IgH* is very similar in B and T cells; (iii) in mature B cells but not in T cells the distal V_H_ regions of both *IgH* alleles position themselves away from active chromatin; and (iv) that these features of the *IgH* locus do not differ between resting and activated lymphocytes.

We confirmed our 4C-seq-based results by microscopy measurements, which did not uncover pronounced differences in PCH proximity between productive and non-productive *IgH* alleles in cycling splenic B cells. Remarkably, our 4C-seq analyses (Figure 6B) also demonstrated that in peripheral resting and activated T cells, both *IgH* alleles are localized to an active compartment. The identified localization in T cells of *IgH* (which is not transcribed in T cells) to active chromatin would be in line with our earlier finding that transcription per se is not necessary to maintain a gene in an active chromatin environment (45), but would be in disagreement with its reported localization near the nuclear lamina. Although the lamina has traditionally been associated with gene silencing (12,44), this region of the nucleus does not exclusively harbor silent genes (46–48).

The finding that productive and non-productive *IgH* alleles occupy similar chromatin environments in resting and cycling splenic B cells adds to the list of similarities between the two: both *IgH* alleles are transcribed in activated splenic B cells, carry similar active chromatin marks, display equivalent RNA polymerase II loading after B cell stimulation (although mRNA of non-productively rearranged alleles is rapidly degraded by non-sense-mediated RNA decay) (19,30) and manifest comparable frequencies of transcription rate-dependent somatic hypermutation in germinal center B cells (49). We conclude that maintenance of allelic exclusion is therefore controlled independent of nuclear location.

It is conceivable that frequent interactions of numerous upstream V_H_ promoters with E_µ_ may interfere with accurate and efficient transcription of the functionally rearranged V_H_ gene that is required in mature B cells and especially in plasma cells in which *IgH* is highly expressed. The observed recruitment of distal V_H_ regions present on both productive and non-productive *IgH* alleles to a less active chromatin environment (Figure 6A) may thus help to silence upstream V_H_ promoters and restrict enhancer activity to the productively rearranged V_H_ promoter element. Accordingly, non-recombined upstream V_H_ segments are thought to be inaccessible, as sense/antisense transcription of these V_H_ segments ceases when *IgH* allelic exclusion is established and is no longer detectable in mature B cells (50).

In summary, using an allele-specific 4C-seq strategy, we analyzed genome-wide contacts made by the productively and non-productively rearranged *IgH* alleles in splenic B cells and the essentially unrearranged *IgH* locus in T cells. Different from published microscopy observations, we find that the overall chromatin environment of these three *IgH* types is similar, except that distal V_H_ regions are in an active chromatin environment in T cells and in a less active chromatin environment in B cells. While it shows that maintenance of allelic exclusion in mature B cells does not depend on nulear positioning, these data do not necessarily suggest that we need to reconsider the importance of nuclear IgH positioning for allelic exclusion during rearrangement in early B cell development. Future allele-specific analyses in pro-B and pre-B cells should reveal the dynamics of chromatin environment during V(D)J recombination events at different stages of B cell development in the bone marrow. 

## AVAILABILITY

Illumina sequencing and micro array expression data have been submitted to the GEO database under accession number: GSE47129.

## SUPPLEMENTARY DATA

Supplementary Data are available at NAR Online: Supplementary Tables 1–13, Supplementary Figures 1–13, Supplementary Methods, and Supplementary References [51–58].

## FUNDING

Funding for open access charge: Dutch Scientific Organization (NWO) [935170621] and a European Research Council Starting Grant [209700, ‘4C’ to W.dL.].

*Conflict of interest statement.* None declared.

## Supplementary Material

Supplementary Data
