# Supplementary material for: Allelic exclusion of the immunoglobulin heavy chain locus is independent of its nuclear localization in mature B cells
Source: Nucleic Acids Res. 2013 Jun 7;41(14):6905–16. doi: 10.1093/nar/gkt491 (PMC3737562; doi:10.1093/nar/gkt491)
Supplement: Supplementary Data [file supp_41_14_6905__index.html]

Allelic exclusion of the immunoglobulin heavy chain locus is independent of its nuclear localization in mature B cells — Allelic exclusion of the immunoglobulin heavy chain locus is independent of its nuclear localization in mature B cells — Supplementary Data 

# Allelic exclusion of the immunoglobulin heavy chain locus is independent of its nuclear localization in mature B cells

## Supplementary Data

files

**Files in this Data Supplement:**

- Supplementary Data - pdf file
