# Supplementary material for: Allelic exclusion of the immunoglobulin heavy chain locus is independent of its nuclear localization in mature B cells
Source: Nucleic Acids Res. 2013 Jun 7;41(14):6905–16. doi: 10.1093/nar/gkt491 (PMC3737562; doi:10.1093/nar/gkt491)

Supplementary information to:

Allelic exclusion of the immunoglobulin heavy chain locus is independent of its nuclear localization in mature B cells

Sjoerd J.B Holwerda[1], Harmen J.G van de Werken[1#], Claudia Ribeiro de Almeida[2#], Ingrid M. Bergen[2], Marjolein J.W. de Bruijn[2], Marjon J. Verstegen[1], Marieke Simonis[1], Erik Splinter[1#], Patrick J. Wijchers[1], Rudi W. Hendriks[2*], Wouter de Laat[1*]

Supplementary Materials and Methods

Mice

Spleens were harvested from F1 hybrid mice (C57Bl/6 x FVB) at 8 to 14 weeks. Fetal brain was isolated from 14.5 dpc mouse embryos. Single cell suspensions, for fetal brains, were prepared by filtration through a 40µm cell strainer (BD biosciences). Mice were bred and kept at specified pathogen free conditions in the Erasmus MC experimental animal facility. All experimental protocols have been reviewed and approved by the Erasmus MC Committee of animal experiments.

Total B and T cell isolation, cell culture and separation of IgM^a and IgM^b expressing B cells

Splenic cell suspensions were prepared and sorted using magnetic beads as previously described (51). Erythrocytes were lysed using Gey's solution. B cells were purified using streptavidin-coupled magnetic beads (Miltenyi Biotec), by negative selection using the following biotinylated antibodies: CD5, CD43, CD138, CD11b, Gr-1 and TER-119 (**Suppl. Table 1.1**). Purified (resting) B cells were *in-vitro* activated, as described in Ref. (18) for 4 days using αCD40 coated plates (20ug/ml; BD Biosciences) and IL-4 (IL-4 50 ng/ml; Peprotech). Splenic T cells were isolated by negative selection using antibodies against B220, NK1.1, CD11b, GR-1 and Ter119 (**Suppl. Table 1.2**). Purified T cells were *in-vitro* activated for 4 days using αCD3 (10µg/ml), αCD28 (10µg/ml) and IL-2 (20µg/ml; R&D systems).

Separation of B cell populations based on IgM allotype expression was done by FACS sorting of MACS-purified fractions of resting B cells (as described above)using antibodies to B220 and CD19, in conjunction with allotype-specific antibodies for IgM^a [FVB] and IgM^b [C57BL/6], as detailed in **Suppl. Table 1.3.**

Mapping 4C single-end and paired-end sequencing data.

The single-end data was mapped, allowing no mismatches, to a database of 4C-seq fragments-ends generated from the mm9/NCBI m37 version of the mouse genome (29). The paired-end sequencing data, however, was first split based on the SNPs (C57Bl/6 vs FVB) detected in the second read (PE2) of the

read-pair and subsequently the first read of the pair (PE1) was mapped as single-end data. If the first read of a pair was at the site of the second restriction enzyme, fragment-ends were used that contain both restriction sites, so called non-blind fragment-ends (26). No mismatches were allowed in the reads.

4C-seq analysis.

4C domainograms were generated as described previously (29,52).

Correlation plots were generated according to the following scheme:

Reads corresponding to *trans* fragment-ends are presumed to reflect single ligation events and are therefore first binarized (29), after which a running window statistic (window size of 500 fragment-ends) was calculated (4C profile). We subsequently, calculated the pairwise Spearman's rank correlation coefficient of the 4C profiles. For the *cis* data, we used a window size of 21 fragment-ends and a z-score was calculated with a running background window of 3001 fragment-ends (25). All the z-scores within 2 Mb of the viewpoint were removed before the pairwise Spearman's rank correlation (r_s) coefficients were calculated. The correlation matrices were clustered based on Euclidean distance of 1-r_s using a hierarchical clustering algorithm with complete linkage. All computations and plotting of the resulting heat maps were carried out with R (53).

Interacting domains were determined according to the following scheme:

To determine which domains are interacting with the viewpoint of interest, we applied a False Discovery Rate (FDR) control (29). In brief, the FDR was calculated after the data was binarized and a binomial one-tailed test was applied on a running window of 100 fragment-ends in *cis* and a background window of 3001. In *trans*, however, the binarized data was transformed with a running window of 500 fragment-ends and a background window based on the number of unique fragment-ends in each chromosome. We permuted (N >=100) the dataset of each chromosome and calculated the FDR based on the *p*-value statistic of the binomial test. Consecutive fragment-ends with a *q*-value < 0.01 and having a maximum gap size of 25 (cis) and 250 (trans) fragment-ends were assigned as a single interacting domain.

RNA extraction, labeling and microarray hybridization.

Expression analysis was done in B and T cells on Affymetrix microarrays (mouse gene st1.0 arrays). RNA was isolated using Trizol® according to manufacturer’s instructions. RNA was labeled according to Affymetrix protocol instructions and hybridized to three independent microarrays per cell type per timepoint according to Affymetrix protocol. Expression data for fetal brain was used from (24).

RNA expression analysis.

Normalized gene expression values were calculated using robust multi-array average (RMA) (54). The

limma package (55) from BioConductor (56) was used for the analysis of differential gene expression in T- and B-cells. Holm's method was used to adjust p-values for multiple testing. Probe sets with an adjusted p-value < 0.05 were selected and their relative expression was plotted in a heat map. The probe sets, as well as, the experiments were clustered after the pairwise Pearson's correlation coefficient (*r*) matrix and the dissimilarity matrix (1-*r*) were calculated. The dissimilarity matrices were used to calculate the Euclidean distances and, subsequently, used for hierarchical clustering with complete linkage. To all 9 clusters, names were assigned based on the expression pattern across the 4 cell types. A Gene Ontology (GO) enrichment analysis was carried out on Entrez genes corresponding to the differentially expressed probe sets. The complete differential expressed gene set and the gene set for each cluster were analyzed with the GO-stats package (57) using a hyper geometrical test for over-representation of genes and a *p*-value threshold-value of 0.001.

Genes that have probe sets on the both Affymetrix gene expression arrays were used to compare the properties of 4C-seq domains. For each gene the median value of the corresponding probe set was assigned, after the gene expression dataset was normalized by subtracting its median value. The gene expression distribution showed a clear bimodal distribution for each RNA-expression experiment. Therefore, the median value of the gene expression dataset was used to discriminate between active or transcribed genes and non-active genes.

RNA-FISH and DNA-FISH

Cells were spotted on poly-L-lysine coated coverslips. RNA FISH experiments were performed as described (27). Nuclei with double CD45 signals were, exclusively used to count IgH RNA signals. DNA FISH experiments were performed as described (27) with minor changes. Denaturing of the DNA in the cells on slides was done for 10' @ 80°C in 50% formamide/2X SSC followed by two washes with 2xSSC at 4°C, after which a denatured probe was applied to the slide for overnight hybridization at 42°C followed by post hybridization washes and microscopic analysis. The following BAC clones were used as probes: For the constant region of the IgH locus, RP23-109B20; for the variable region of the IgH locus, RP24-376H17 (kindly provided by M. Busslinger); for *CD45*, RP24-371I24; for γ-satelites a pBleuscript plasmid containing 8 copies of repetitive γ-satelite sequence was used as described (18,58) (kindly provided by J.A. Skok). For the verification of the 4C data the following BAC clones were used. For two interactions in B cells RP23-215D11 and RP23-432F6 were used. For interactions in fetal brain, RP23-69J9 was used. Generation of the probes was done as described (25). Specificity of the probes was verified on metaphase spreads of mouse ES cells (data not shown). 3D distance measurements were done for at least 50 nuclei per data point, using Image J software. The active allele is defined as the least

contracted allele. The other allele is defined as the inactive allele. Distances towards peri-centromeric heterochromatin are measured as the minimal distance of the IgH locus towards the edge of the γ-satellite signal.

Supplemental references

18. Skok, J.A., Brown, K.E., Azuara, V., Caparros, M.L., Baxter, J., Takacs, K., Dillon, N., Gray, D., Perry, R.P., Merkenschlager, M. *et al.* (2001) Nonequivalent nuclear location of immunoglobulin alleles in B lymphocytes. *Nature immunology*, **2**, 848-854.
24. Simonis, M., Klous, P., Splinter, E., Moshkin, Y., Willemsen, R., de Wit, E., van Steensel, B. and de Laat, W. (2006) Nuclear organization of active and inactive chromatin domains uncovered by chromosome conformation capture-on-chip (4C). *Nature genetics*, **38**, 1348-1354.
25. Splinter, E., de Wit, E., Nora, E.P., Klous, P., van de Werken, H.J., Zhu, Y., Kaaij, L.J., van Ijcken, W., Gribnau, J., Heard, E. *et al.* (2011) The inactive X chromosome adopts a unique three-dimensional conformation that is dependent on Xist RNA. *Genes & development*, **25**, 1371-1383.
26. van de Werken, H.J., Landan, G., Holwerda, S.J., Hoichman, M., Klous, P., Chachik, R., Splinter, E., Valdes-Quezada, C., Oz, Y., Bouwman, B.A. *et al.* (2012) Robust 4C-seq data analysis to screen for regulatory DNA interactions. *Nature methods*, **9**, 969-972.
27. Chaumeil, J., Le Baccon, P., Wutz, A. and Heard, E. (2006) A novel role for Xist RNA in the formation of a repressive nuclear compartment into which genes are recruited when silenced. *Genes & development*, **20**, 2223-2237.
29. van de Werken, H.J., de Vree, P.J., Splinter, E., Holwerda, S.J., Klous, P., de Wit, E. and de Laat, W. (2012) 4C technology: protocols and data analysis. *Methods in enzymology*, **513**, 89-112.
51. Kil, L.P., de Bruijn, M.J., van Nimwegen, M., Corneth, O.B., van Hamburg, J.P., Dingjan, G.M., Thaiss, F., Rimmelzwaan, G.F., Elewaut, D., Delsing, D. *et al.* (2012) Btk levels set the threshold for B-cell activation and negative selection of autoreactive B cells in mice. *Blood*, **119**, 3744-3756.
52. de Wit, E., Braunschweig, U., Greil, F., Bussemaker, H.J. and van Steensel, B. (2008) Global chromatin domain organization of the Drosophila genome. *PLoS genetics*, **4**, e1000045.
53. R Development Core Team. (2010). R: A language and environment for statistical computing. Vienna, Austria: R Foundation for Statistical Computing.
54. Irizarry, R.A., Bolstad, B.M., Collin, F., Cope, L.M., Hobbs, B. and Speed, T.P. (2003) Summaries of Affymetrix GeneChip probe level data. *Nucleic acids research*, **31**, e15.
55. Smyth, G.K., Michaud, J. and Scott, H.S. (2005) Use of within-array replicate spots for assessing differential expression in microarray experiments. *Bioinformatics*, **21**, 2067-2075.
56. Gentleman, R.C., Carey, V.J., Bates, D.M., Bolstad, B., Dettling, M., Dudoit, S., Ellis, B., Gautier, L., Ge, Y., Gentry, J. *et al.* (2004) Bioconductor: open software development for computational biology and bioinformatics. *Genome biology*, **5**, R80.
57. Falcon, S. and Gentleman, R. (2007) Using GOstats to test gene lists for GO term association. *Bioinformatics*, **23**, 257-258.
58. Brown, K.E., Guest, S.S., Smale, S.T., Hahm, K., Merkenschlager, M. and Fisher, A.G. (1997) Association of transcriptionally silent genes with Ikaros complexes at centromeric heterochromatin. *Cell*, **91**, 845-854.

Legends to supplemental figures

Supplementary table 1. Antibodies used in the MACS on T (**S1.1**) and B cells (**S1.2**) and the subsequent FACS where B cells were separated based on the haplotypic origin of their B cell receptor (**S1.3**).

Supplementary table 2. Gene expression changes between (D4 / D0) confirm *in-vitro* activation of resting, splenic B cells. Absolute fold change is calculated as the average expression, of three independent microarray experiments per cell type, of the activated B cells over the resting B cells.

Supplementary table 3-11. Representation of the gene ontology analysis that was done on the 9 clusters as identified in the gene expression analysis of the resting and activated B and T cells (see supplemental figure 10). Supplementary table 3-11 depict the list of biological processes that were found in the GO analysis for gene clusters 1-9, respectively. The lists of genes are sorted based on the P-values, from small to large.

Supplementary table 12. Sequence of the 4C primers that were used in the single end 4Cseq experiment (S12a) and the paired-end 4Cseq experiment (S12b).

Supplementary table 13. Genomic positions of the SNPs that are used to assign a 4C capture to the C57Bl/6 or the FVB allele in the paired-end 4Cseq strategy.

Supplemental figure 1. Lack of RNA signal after RNase treatment.
Representative RNA FISH picture for RNase treated resting B cells.

Supplemental figure 2. Single end allele specific 4C strategy.
Schematic representation of a 4C HindIII/DpnII fragment of a C57Bl/6 and a FVB allele. A SNP between the two alleles creates an additional DpnII restriction site (*red scissor pair*) in the FVB allele which prohibits the formation of a 4C PCR product using the inverse PCR primer 1 and 2.

Supplemental figure 3. Quality control of the 4C experiments.
Cumulative read distribution of all experiments plotted as the percentage of captured reads in different binned regions. Distance of the regions are relative to the viewpoints.

Supplemental figure 4. Local contact profiles at the FVB allele.

(A,B) Contact profiles of the *IgH* locus in resting **(A)** and activated **(B)** B cells, from left to right looking from: *3'RR* (i), *Cγ3* (ii) and $d\text{-}V_H$ (iii). **(C,D)** Zoom-in at 3'border (*dotted black* line) of *IgH* locus in resting **(C)** and activated **(D)** B cells, from left to right looking from: *3'RR* (i), *Cγ3* (ii) and $d\text{-}V_H$ (iii). The black line above the tracks indicates the region where the productive allele in B cells has only few interactions compared to the other alleles. **(E)** Zoom-in at 5'border (*dotted black line*) of IgH locus in resting (i) and activated (ii) B cells, looking from 4C viewpoint $d\text{-}V_H$.

Supplemental figure 5. Topological and recombination borders defined by HiC and 4C.
Contacts around the IgH locus are shown from top to bottom as Hi-C data in ESCs (i), $RAG1^{-/-}$ pro B cells (ii) and a G1 arrested pre-B cell line (iii). 4C data (iv) for the *IgH* locus looking from the $d\text{-}V_H$ 4C viewpoint in resting B cells. Top to bottom: the productive (red) and non-productive B cell allele (blue), T cells (blue) and fetal brain (black). The black bar depicts the region that is recombined in the preB cell line (iii) and on the productive allele (iv). The shaded region depicts the topological domain spanning the IgH locus.

Supplemental figure 6. Difference in 4C contacts at the proximal V region.
Raw 4C contact data looking from the $d\text{-}V_H$ 4C viewpoint in resting (i) and activated (ii) B cells. Contacts for the productive and the non-productive B cell alleles are shown in red and grey, respectively. Purple colored 4C signals depict saturation of the signal given the maximum value of the Y-axis.

Supplemental figure 7. *Cis* contacts of the IgH locus at the FVB allele.
(A-F) Domainograms showing chromosome-wide interactions of the FVB allele, in resting **(A)** or activated B cells **(B)** looking from the *3'RR* 4C viewpoint; in resting **(C)** or activated B cells **(D)** looking from the *Cγ3* viewpoint; in resting **(E)** or activated B cells **(F)** looking from the $d\text{-}V_H$ viewpoint. Different alleles are depicted below each other, from top to bottom: P = productive allele in B cells, NP = non-productive allele in B cells, T = T cells, FB = fetal brain. Red arrows depict the 4C viewpoints. Significance of the interactions is indicated by the range in color used in the domainogram as depicted in the legend: black is low significance (P = 1) and yellow represents high significance ($P = 10^{-10}$) of interaction.

Supplemental figure 8. Verification of 4C by DNA FISH.
(A) Schematic representation of the probe positions. Green arrows depict the positions of the probes used to verify the 4C interaction, the red arrow depicts the position of the probe used as a negative control, the black arrow depicts probe that is used to visualize the *IgH* locus. **(B)** Representative pictures of the DNA

FISH, showing an interaction A (i) and the negative control (ii). Scale bar represents 4μm. **(C)** Cumulative fractions of cells plotted as a function of the distance between the test probe and the probe for the *IgH* locus. A minimum of 50 cells was counted per probe set.

Supplemental figure 9. *Cis* contacts of the *IgH* locus at the C57BL/6 allele.

(A-F) Domainograms showing chromosome-wide interactions of the C57Bl/6 allele, in resting **(A)** or activated B cells **(B)** looking from the *Cγ3* 4C viewpoint; in resting **(C)** or activated B cells **(D)** looking from the $d\text{-}V_H$ viewpoint; in resting **(E)** or activated B cells **(F)** looking from the *Up-IgH* viewpoint. Different alleles are depicted below each other, from top to bottom: P = productive allele in B cells, NP = non-productive allele in B cells, T = T cells, FB = fetal brain. Red arrows depict the 4C viewpoints. Significance of the interactions is indicated by the range in color used in the domainogram as depicted in the legend: black is low significance (P = 1) and yellow represents high significance ($P = 10^{-10}$) of interaction.

Supplemental figure 10. Correlation of *cis* contacts.

(A,B) Correlation plots of the interactions looking from the *Cγ3* viewpoint in resting **(A)** or activated **(B)** B cells (window size 21). The numbers represent the Spearman rank correlation coefficient, colors range from linear anti-correlation (black) to linear correlation (dark red). Cell types and the alleles are depicted along the X and Y-axis; B cells productive (P), B cell non-productive (NP), T cell (T), fetal brain (FB), C57BL/6 allele (Bl6) and FVB allele (FVB). **(C,D)** Correlation plots of the interactions looking from the $d\text{-}V_H$ viewpoint in resting **(C)** and activated **(D)** B cells (window size 21). **(E,F)** Correlation plots of the interactions looking from the *Up-IgH* viewpoint in resting **(E)** and activated **(F)** B cells.

Supplemental figure 11. Correlation of *trans* contacts.

(A,B) Correlation plot of the interactions looking from the *3'RR* viewpoint in resting **(A)** and activated **(B)** B cells (window size 21). The numbers represent the Spearman rank correlation coefficient, colors range from linear anti-correlation (black) to linear correlation (dark red). Cell types and the alleles are depicted along the X and Y-axis; B cells productive (P), B cell non-productive (NP), T cell (T), fetal brain (FB), C57BL/6 allele (Bl6) and FVB allele (FVB). **(C,D)** Correlation plot of the interactions looking from the *Cγ3* viewpoint in resting **(C)** and activated **(D)** B cells. (E,F) Correlation plot of the interactions looking from the $d\text{-}V_H$ viewpoint in resting **(E)** and activated **(F)** B cells. **(G,H)** Correlation plot of the interactions looking from the *Up-IgH* viewpoint in resting **(G)** and activated **(H)** B cells.

Supplemental figure 12. (A). Correlation plot of the interactions of C57BL/6 viewpoints in *cis*, in resting B cells. The X-axis shows the productive (P) and the non-productive (NP) C57Bl/6 alleles looking from the four 4C viewpoints. The *IgH* locus with the position of the 4C viewpoints is drawn to scale below the X-axis. The numbers represent the Spearman rank correlation coefficients, colors range from linear anti-correlation (black) to linear correlation (dark red). The dotted line represent the topological border that is present between the d-V_H and the *Up-IgH* 4C viewpoints. **(B)** Correlation plot of the interactions of C57BL/6 viewpoints in *cis* in resting T cells.

Supplemental figure 13. Clustering of gene expression data in resting and activated B and T cells. Hierarchical clustering of microarray data in resting B and T cells. On the horizontal axis the tissues and the microarray experiments are depicted, highlighted in colors. The numbers refer to the biological triplo experiments per cell type. The vertical axis shows the names of the 9 different clusters. The name of a cluster depicts the tissue and the up/down regulation of the genes that define the specific cluster.

Supplemental table 1.
Antibodies used in FACS/MACS

Table S1.1 MACS on B cells

Name	Company	Product number
CD5	BD	553019
CD43	ebioscience	13-0431-85
CD138	BD	553713
CD11b	BD	553309
Ly-6G (GR-1)	ebioscience	13-5931-85
TER-119	ebioscience	13-5921-85

Table S1.2 MACS on T cells

Name	Company	Product number
CD45R/B220	BD	553085
NK-1.1	BD	553163
CD11b	BD	553309
GR-1	ebioscience	13-5931-85
TER-119	ebioscience	13-5921-85

Table S1.3 Allotypic FACS on B cells

Name	Company	Product number
CD19 PerCP-Cy5.5	ebioscience	45-0193-82
CD45R (B220) FITC	ebioscience	11-0452-86
IgM$_a$	BD	553517
IgMb	BD	553519
Streptavidin APC	ebioscience	17-4317-82

Supplemental table 2.

EntrezID	gene symbols	gene description	Fold Change (D4/ D0)	P value
11628	Aicda	activation-induced cytidine deaminase	79.34	0
16192	Il5ra	interleukin 5 receptor, alpha	15.45	0
12505	Cd44	CD44 antigen	5.06	0
14102	Fas	Fas (TNF receptor superfamily member 6)	7.31	0
12444	Ccnd2	cyclin D2	7.06	0
17869	Myc	myelocytomatosis oncogene	1.99	0

Supplemental Table 3a.

Upregulated genes in cycling B and T cells (day 4)

GOBPID	Pvalue	OddsRatio	ExpCount	Count	Size	Term
GO:0000278	0	8.54506	23	131	493	mitotic cell cycle
GO:0048285	0	11.77863	15	107	313	organelle fission
GO:0000279	0	9.07895	17	106	377	M phase
GO:0051301	0	9.54178	16	101	344	cell division
GO:0022402	0	6.97876	17	87	394	cell cycle process
GO:0007067	0	12.09882	8	61	175	mitosis
GO:0006807	0	2.26305	219	370	4649	nitrogen compound metabolic process
GO:0071840	0	2.32487	165	300	3515	cellular component organization or biogenesis
GO:0007059	0	13.50008	5	38	98	chromosome segregation
GO:0006281	0	5.56945	15	66	321	DNA repair
GO:0006139	0	2.1394	192	320	4185	nucleobase-containing compound metabolic process
GO:0051325	0	6.95954	9	47	190	interphase
GO:0071842	0	2.23124	119	219	2601	cellular component organization at cellular level
GO:0006323	0	10.96241	5	33	96	DNA packaging
GO:0007017	0	5.67163	11	49	235	microtubule-based process
GO:0045786	0	5.03255	13	54	282	negative regulation of cell cycle
GO:0051276	0	3.44368	27	80	586	chromosome organization
GO:0044249	0	1.95967	171	275	3740	cellular biosynthetic process
GO:0010564	0	6.69623	7	34	144	regulation of cell cycle process
GO:0006310	0	6.07653	8	36	160	DNA recombination
GO:0006260	0	5.90243	7	34	158	DNA replication
GO:0006259	0	6.31407	6	31	145	DNA metabolic process
GO:0009161	0	18.29423	2	16	34	ribonucleoside monophosphate metabolic process
GO:0044281	0	2.29552	54	110	1205	small molecule metabolic process
GO:0007049	0	4.24271	11	40	284	cell cycle
GO:0006270	0	41.00556	1	12	18	DNA-dependent DNA replication initiation
GO:0009124	0	19.27525	1	15	31	nucleoside monophosphate biosynthetic process
GO:0051321	0	5.47345	7	30	144	meiotic cell cycle
GO:0009126	0	19.16923	1	14	29	purine nucleoside monophosphate metabolic process
GO:0006950	0	1.90543	94	158	1987	response to stress
GO:0044238	0	2.6557	32	73	960	primary metabolic process
GO:0006695	0	14.0138	2	15	37	cholesterol biosynthetic process
GO:0000724	0	12.19019	2	16	43	double-strand break repair via homologous recombination
GO:0031570	0	7.64185	3	20	74	DNA integrity checkpoint
GO:0000082	0	6.40178	4	22	93	G1/S transition of mitotic cell cycle
GO:0009059	0	1.66461	158	231	3356	macromolecule biosynthetic process
GO:0016125	0	5.79801	5	23	105	sterol metabolic process
GO:0006333	0	6.76234	4	20	81	chromatin assembly or disassembly
GO:0007010	0	2.39708	33	71	699	cytoskeleton organization
GO:0006261	0	10.41903	2	15	45	DNA-dependent DNA replication
GO:0031023	0	8.32886	3	17	59	microtubule organizing center organization
GO:0007093	0	7.56455	3	18	67	mitotic cell cycle checkpoint
GO:0000070	0	14.5914	1	12	29	mitotic sister chromatid segregation
GO:0019320	0	6.99212	3	18	71	hexose catabolic process
GO:0007076	0	54.44075	1	8	11	mitotic chromosome condensation
GO:0042180	0	2.31844	33	70	709	cellular ketone metabolic process
GO:0019752	0	2.34955	32	68	680	carboxylic acid metabolic process
GO:0006753	0	2.07829	47	89	1002	nucleoside phosphate metabolic process
GO:0006334	0	8.80246	2	15	50	nucleosome assembly
GO:0009259	0	2.28875	34	70	717	ribonucleotide metabolic process
GO:0009152	0	7.16838	3	17	66	purine ribonucleotide biosynthetic process
GO:0043412	0	1.70518	107	165	2282	macromolecule modification
GO:0019538	0	1.58871	164	231	3479	protein metabolic process
GO:0006793	0	1.78464	80	130	1703	phosphorus metabolic process
GO:0006096	0	7.70006	3	15	55	glycolysis
GO:0034453	0	11.17363	2	12	34	microtubule anchoring
GO:0090407	0	3.28595	12	34	249	organophosphate biosynthetic process
GO:0009056	0	1.78054	79	128	1707	catabolic process
GO:0034502	0	15.73039	1	10	23	protein localization to chromosome
GO:0034622	0	2.66294	19	46	407	cellular macromolecular complex assembly
GO:0033043	0	2.58747	21	48	436	regulation of organelle organization

Supplemental Figure 3b.

Upregulated genes in cycling B and T cells (day 4)

GOBPID	Pvalue	OddsRatio	ExpCount	Count	Size	Term
GO:0031572	0	11.84848	1	11	30	G2/M transition DNA damage checkpoint
GO:0000090	0	122.23425	0	6	7	mitotic anaphase
GO:0071824	0	5.36608	4	18	87	protein-DNA complex subunit organization
GO:0009168	0	23.43048	1	8	15	purine ribonucleoside monophosphate biosynthetic process
GO:0044260	0	1.53359	181	242	4444	cellular macromolecule metabolic process
GO:0022607	0	1.83568	61	102	1287	cellular component assembly
GO:0009411	0	5.14166	4	18	90	response to UV
GO:0048519	0	1.56085	137	194	2918	negative regulation of biological process
GO:0016053	0	3.08562	12	32	247	organic acid biosynthetic process
GO:0046483	0	1.88903	51	88	1084	heterocycle metabolic process
GO:0051656	0	5.66519	3	16	74	establishment of organelle localization
GO:0046040	0	61.11381	0	6	8	IMP metabolic process
GO:0051988	0	61.11381	0	6	8	regulation of attachment of spindle microtubules to kinetochore
GO:0009163	0	11.35775	1	10	28	nucleoside biosynthetic process
GO:0000079	0	6.37934	3	14	59	regulation of cyclin-dependent protein kinase activity
GO:0031145	0	23.78761	1	7	13	anaphase-promoting complex-dependent proteasomal ubiquitin-dependent protein catabolic process
GO:0032465	0	12.25477	1	9	24	regulation of cytokinesis
GO:1901135	0	1.84115	51	87	1087	carbohydrate derivative metabolic process
GO:0016052	0	4.11627	6	20	120	carbohydrate catabolic process
GO:0007088	0	5.49526	3	15	71	regulation of mitosis
GO:0044237	0	2.90157	12	31	396	cellular metabolic process
GO:0051052	0	3.25354	9	26	191	regulation of DNA metabolic process
GO:0043067	0	1.81461	52	88	1114	regulation of programmed cell death
GO:0032392	0	8.03611	2	11	39	DNA geometric change
GO:0006006	0	3.43743	8	24	168	glucose metabolic process
GO:0033261	0	9.29069	2	10	32	regulation of S phase
GO:0009113	0	101.74945	0	5	6	purine nucleobase biosynthetic process
GO:0022900	0	4.30136	5	18	104	electron transport chain
GO:0050000	0	13.60354	1	8	20	chromosome localization
GO:0005996	0	3.08079	10	27	208	monosaccharide metabolic process
GO:0070925	0	4.62463	4	16	87	organelle assembly
GO:0009058	0	4.76804	4	15	94	biosynthetic process
GO:0009165	0	2.96522	10	27	216	nucleotide biosynthetic process
GO:0008219	0	1.68084	67	104	1417	cell death
GO:0006461	0	1.97653	33	61	707	protein complex assembly
GO:0008652	0	4.43642	4	16	90	cellular amino acid biosynthetic process
GO:0072528	0	11.65891	1	8	22	pyrimidine-containing compound biosynthetic process
GO:0007018	0	3.79169	6	19	122	microtubule-based movement
GO:0006268	0	50.87196	0	5	7	DNA unwinding involved in replication
GO:0006177	0	Inf	0	4	4	GMP biosynthetic process
GO:0006189	0	Inf	0	4	4	'de novo' IMP biosynthetic process
GO:0006694	0.00001	3.71906	6	19	124	steroid biosynthetic process
GO:0046034	0.00001	2.64874	13	31	273	ATP metabolic process
GO:0009628	0.00001	2.0513	28	53	592	response to abiotic stimulus
GO:0007127	0.00001	5.32392	3	13	63	meiosis I
GO:0071777	0.00001	20.36685	1	6	12	positive regulation of cell cycle cytokinesis
GO:0016310	0.00001	2.64182	12	30	274	phosphorylation
GO:0055114	0.00001	1.93898	33	59	705	oxidation-reduction process
GO:0009142	0.00001	5.58014	3	12	56	nucleoside triphosphate biosynthetic process
GO:0051289	0.00001	5.58014	3	12	56	protein homotetramerization
GO:0006163	0.00001	1.83307	40	69	858	purine nucleotide metabolic process
GO:0008608	0.00001	34.13519	0	5	8	attachment of spindle microtubules to kinetochore
GO:0009112	0.00001	9.5999	1	8	25	nucleobase metabolic process
GO:0090307	0.00001	17.45635	1	6	13	spindle assembly involved in mitosis
GO:0006760	0.00002	11.88993	1	7	19	folic acid-containing compound metabolic process
GO:0031324	0.00002	1.67276	58	90	1224	negative regulation of cellular metabolic process
GO:0008637	0.00002	5.22311	3	12	59	apoptotic mitochondrial changes
GO:0010212	0.00002	4.1545	4	15	89	response to ionizing radiation
GO:0006974	0.00002	3.68908	5	17	118	response to DNA damage stimulus

Supplemental Table 4.

Upregulated in cycling T cells (day 4)

GOBPID	Pvalue	OddsRatio	ExpCount	Count	Size	Term
GO:0016265	0	3.89883	12	37	1422	death
GO:0010740	0	7.27351	3	18	349	positive regulation of intracellular protein kinase cascade
GO:0031347	0	7.35463	2	15	283	regulation of defense response
GO:0006954	0	7.31752	2	15	297	inflammatory response
GO:0030003	0	6.6614	3	16	333	cellular cation homeostasis
GO:0051179	0	2.59032	32	63	3942	localization
GO:0043067	0	3.7224	9	29	1114	regulation of programmed cell death
GO:0055082	0	4.69064	5	21	624	cellular chemical homeostasis
GO:0006950	0	2.97687	16	40	1987	response to stress
GO:0035556	0	3.25275	12	33	1463	intracellular signal transduction
GO:0048584	0	3.73271	8	26	981	positive regulation of response to stimulus
GO:0008284	0	4.51236	5	20	613	positive regulation of cell proliferation
GO:0032101	0	5.99022	3	15	343	regulation of response to external stimulus
GO:0007204	0	8.7299	1	11	173	elevation of cytosolic calcium ion concentration
GO:0050801	0	4.3531	5	20	634	ion homeostasis
GO:0055065	0	6.1002	3	14	313	metal ion homeostasis
GO:0032943	0	7.93851	2	11	189	mononuclear cell proliferation
GO:0072507	0	6.3833	2	13	277	divalent inorganic cation homeostasis
GO:0050864	0	12.98149	1	8	86	regulation of B cell activation
GO:0006810	0	2.42389	26	51	3180	transport
GO:0051094	0	4.02704	6	20	682	positive regulation of developmental process
GO:0023056	0	3.84812	6	21	751	positive regulation of signaling
GO:0009966	0	2.93753	13	32	1548	regulation of signal transduction
GO:0042110	0	5.99174	2	13	294	T cell activation
GO:0002376	0	3.10477	10	28	1264	immune system process
GO:2000026	0	3.24303	9	26	1117	regulation of multicellular organismal development
GO:0050728	0	14.44165	1	7	68	negative regulation of inflammatory response
GO:0060548	0	4.0246	5	19	645	negative regulation of cell death
GO:0070887	0	3.1964	9	26	1132	cellular response to chemical stimulus
GO:0042592	0	3.19028	9	26	1134	homeostatic process
GO:0060401	0	13.3441	1	7	73	cytosolic calcium ion transport
GO:0043066	0	3.99656	5	18	612	negative regulation of apoptotic process
GO:0030334	0	4.91109	3	14	384	regulation of cell migration
GO:0009893	0	2.6637	15	34	1812	positive regulation of metabolic process
GO:0010647	0	3.62818	6	20	752	positive regulation of cell communication
GO:0001525	0	5.26738	3	13	332	angiogenesis
GO:0050670	0	8.22345	1	9	148	regulation of lymphocyte proliferation
GO:0010035	0	5.65657	2	12	285	response to inorganic substance
GO:0032940	0	4.0272	5	17	571	secretion by cell
GO:0070663	0.00001	7.88069	1	9	154	regulation of leukocyte proliferation
GO:0002694	0.00001	5.51312	2	12	292	regulation of leukocyte activation
GO:0051209	0.00001	15.31893	0	6	55	release of sequestered calcium ion into cytosol
GO:0051282	0.00001	15.31893	0	6	55	regulation of sequestering of calcium ion
GO:0002237	0.00001	6.6408	2	10	202	response to molecule of bacterial origin
GO:0045785	0.00001	8.94427	1	8	121	positive regulation of cell adhesion
GO:0006874	0.00001	5.86852	2	11	251	cellular calcium ion homeostasis
GO:0050776	0.00001	4.92181	3	13	354	regulation of immune response
GO:0070838	0.00001	5.3202	2	12	302	divalent metal ion transport
GO:0051270	0.00001	4.45585	3	14	421	regulation of cellular component movement
GO:0030890	0.00001	20.06598	0	5	36	positive regulation of B cell proliferation
GO:0033993	0.00001	20.06598	0	5	36	response to lipid
GO:0051241	0.00001	5.08844	3	12	315	negative regulation of multicellular organismal process
GO:0040012	0.00001	4.36809	4	14	429	regulation of locomotion
GO:0002700	0.00001	13.1634	1	6	63	regulation of production of molecular mediator of immune response
GO:0051238	0.00001	18.84789	0	5	38	sequestering of metal ion
GO:0001819	0.00002	6.79357	1	9	177	positive regulation of cytokine production
GO:0051247	0.00002	3.46968	6	18	699	positive regulation of protein metabolic process
GO:0044093	0.00002	3.01306	8	22	993	positive regulation of molecular function
GO:0097285	0.00002	5.20821	2	11	281	cell-type specific apoptotic process
GO:0032800	0.00002	29.10512	0	4	21	receptor biosynthetic process
GO:0061298	0.00002	29.10512	0	4	21	retina vasculature development in camera-type eye
GO:0050867	0.00003	6.3724	2	9	188	positive regulation of cell activation
GO:0043065	0.00003	3.80005	4	15	527	positive regulation of apoptotic process
GO:0048731	0.00003	2.16325	24	44	2928	system development
GO:0001568	0.00004	3.9226	4	14	475	blood vessel development
GO:0051707	0.00004	3.9226	4	14	475	response to other organism
GO:0002673	0.00004	14.8021	0	5	47	regulation of acute inflammatory response
GO:0009894	0.00004	3.68174	4	15	543	regulation of catabolic process
GO:0042327	0.00005	3.86248	4	14	482	positive regulation of phosphorylation
GO:0010942	0.00005	3.64622	5	15	548	positive regulation of cell death
GO:0071248	0.00005	14.1278	0	5	49	cellular response to metal ion
GO:0009653	0.00005	2.39548	15	31	1793	anatomical structure morphogenesis
GO:0010562	0.00005	3.80414	4	14	489	positive regulation of phosphorus metabolic process
GO:0045124	0.00006	22.48446	0	4	26	regulation of bone resorption
GO:0051251	0.00006	6.54889	1	8	162	positive regulation of lymphocyte activation
GO:0048519	0.00006	2.21842	20	38	2603	negative regulation of biological process
GO:0007260	0.00006	13.51214	0	5	51	tyrosine phosphorylation of STAT protein
GO:0048771	0.00006	7.70614	1	7	121	tissue remodeling
GO:0008285	0.0001	3.78556	4	13	454	negative regulation of cell proliferation

Supplemental Table 5.

Upregulated genes in resting T cells (day 0)

GOBPID	Pvalue	OddsRatio	ExpCount	Count	Size	Term
GO:0006793	0	2.9123	20	48	1703	phosphorus metabolic process
GO:0016310	0	2.98903	16	42	1427	phosphorylation
GO:0045321	0	4.42376	6	23	510	leukocyte activation
GO:0002764	0	7.92075	2	12	149	immune response-regulating signaling pathway
GO:0001817	0	4.84298	4	17	339	regulation of cytokine production
GO:0050790	0	2.67374	17	39	1451	regulation of catalytic activity
GO:0034097	0	4.89087	4	16	315	response to cytokine stimulus
GO:0006952	0	3.34561	8	25	724	defense response
GO:0002520	0	3.65381	7	22	581	immune system development
GO:0019221	0	6.4876	2	12	179	cytokine-mediated signaling pathway
GO:0002684	0	4.13096	5	18	418	positive regulation of immune system process
GO:0006955	0	3.49269	7	22	606	immune response
GO:0007243	0	3.27326	8	24	707	intracellular protein kinase cascade
GO:0030097	0	3.71138	6	20	517	hemopoiesis
GO:0048584	0	2.86865	11	29	981	positive regulation of response to stimulus
GO:0030155	0.00001	4.97898	3	13	249	regulation of cell adhesion
GO:0002253	0.00001	5.84812	2	11	180	activation of immune response
GO:0042110	0.00001	4.53053	3	14	294	T cell activation
GO:0023052	0.00001	1.87004	56	85	4850	signaling
GO:0007154	0.00001	1.84918	57	86	4960	cell communication
GO:0071310	0.00001	2.84771	10	25	841	cellular response to organic substance
GO:2000503	0.00001	130.99315	0	3	5	positive regulation of natural killer cell chemotaxis
GO:0030098	0.00001	4.95649	3	12	230	lymphocyte differentiation
GO:0032651	0.00002	18.34005	0	5	29	regulation of interleukin-1 beta production
GO:0036211	0.00002	2.11517	25	47	2202	protein modification process
GO:0051716	0.00002	1.79937	61	90	5346	cellular response to stimulus
GO:0031325	0.00002	2.24246	19	39	1699	positive regulation of cellular metabolic process
GO:0050727	0.00002	5.73627	2	10	166	regulation of inflammatory response
GO:0031401	0.00003	3.22161	6	19	559	positive regulation of protein modification process
GO:0032729	0.00003	16.29971	0	5	32	positive regulation of interferon-gamma production
GO:0072678	0.00004	26.97812	0	4	17	T cell migration
GO:0050789	0.00004	1.72679	98	128	8561	regulation of biological process
GO:0019220	0.00004	2.689	10	24	849	regulation of phosphate metabolic process
GO:0048247	0.00005	25.0498	0	4	18	lymphocyte chemotaxis
GO:0032496	0.00005	5.19828	2	10	182	response to lipopolysaccharide
GO:0002429	0.00005	8.05497	1	7	84	immune response-activating cell surface receptor signaling pathway
GO:0035556	0.00006	2.8586	8	21	756	intracellular signal transduction
GO:0051247	0.00006	2.84293	8	21	699	positive regulation of protein metabolic process
GO:0030335	0.00006	4.60741	3	11	225	positive regulation of cell migration
GO:0002697	0.00006	5.0501	2	10	187	regulation of immune effector process
GO:0006140	0.00006	3.51825	5	15	401	regulation of nucleotide metabolic process
GO:0048585	0.00007	2.81708	8	21	705	negative regulation of response to stimulus
GO:0001932	0.00007	2.80009	8	21	709	regulation of protein phosphorylation
GO:0002758	0.00008	9.45982	1	6	62	innate immune response-activating signal transduction
GO:0032612	0.00008	12.93914	0	5	39	interleukin-1 production
GO:0010820	0.00008	52.38904	0	3	8	positive regulation of T cell chemotaxis
GO:0009607	0.00008	3.16072	6	17	506	response to biotic stimulus
GO:2000401	0.00009	20.62601	0	4	21	regulation of lymphocyte migration
GO:0051272	0.0001	4.36	3	11	237	positive regulation of cellular component movement
GO:0045089	0.0001	7.2086	1	7	93	positive regulation of innate immune response

Supplemental Table 6

Upregulated genes in resting and cycling T cells (day 0 and day 4)

GOBPID	Pvalue	OddsRatio	ExpCount	Count	Size	Term
GO:0002429	0	23.37377	1	16	84	immune response-activating cell surface receptor signaling pathway
GO:0002682	0	5.36871	7	32	659	regulation of immune system process
GO:0002521	0	7.52742	4	23	334	leukocyte differentiation
GO:0048583	0	3.37403	21	57	1983	regulation of response to stimulus
GO:0050852	0	55.12212	0	9	25	T cell receptor signaling pathway
GO:0006955	0	5.43217	7	30	606	immune response
GO:0002764	0	11.90977	2	16	149	immune response-regulating signaling pathway
GO:0010646	0	3.62956	15	45	1398	regulation of cell communication
GO:0048534	0	5.26883	6	27	554	hemopoietic or lymphoid organ development
GO:0002253	0	9.64278	2	16	180	activation of immune response
GO:0001775	0	7.89431	3	18	266	cell activation
GO:0023051	0	3.06449	19	49	1798	regulation of signaling
GO:0002696	0	8.99327	2	15	179	positive regulation of leukocyte activation
GO:0050865	0	6.44446	3	19	313	regulation of cell activation
GO:0048522	0	2.52074	32	64	2933	positive regulation of cellular process
GO:0045058	0	60.06281	0	5	13	T cell selection
GO:0050850	0	31.55611	0	6	24	positive regulation of calcium-mediated signaling
GO:0033077	0	12.72067	1	8	68	T cell differentiation in thymus
GO:0046632	0	12.72067	1	8	68	alpha-beta T cell differentiation
GO:0001816	0	4.57853	4	17	382	cytokine production
GO:0051249	0	9.52811	1	9	103	regulation of lymphocyte activation
GO:0046649	0	7.94776	1	10	144	lymphocyte activation
GO:0009967	0	3.56267	7	22	663	positive regulation of signal transduction
GO:0019932	0	7.80898	1	10	133	second-messenger-mediated signaling
GO:0009968	0	3.74291	6	20	549	negative regulation of signal transduction
GO:0046651	0	17.34349	0	6	40	lymphocyte proliferation
GO:0070661	0	16.84175	0	6	41	leukocyte proliferation
GO:0045060	0.00001	46.89461	0	4	12	negative thymic T cell selection
GO:0042102	0.00001	11.45831	1	7	65	positive regulation of T cell proliferation
GO:0010941	0.00001	2.70158	12	30	1154	regulation of cell death
GO:0042981	0.00001	2.72538	12	29	1103	regulation of apoptotic process
GO:0007166	0.00001	2.06487	33	58	3077	cell surface receptor signaling pathway
GO:0043368	0.00001	41.68192	0	4	13	positive T cell selection
GO:0006796	0.00001	2.34619	18	38	1703	phosphate-containing compound metabolic process
GO:0050870	0.00001	13.31399	1	6	50	positive regulation of T cell activation
GO:0048519	0.00001	2.04386	31	55	2918	negative regulation of biological process
GO:0050670	0.00003	6.18264	2	9	148	regulation of lymphocyte proliferation
GO:0006464	0.00003	2.11141	24	44	2202	cellular protein modification process
GO:0042035	0.00004	8.51135	1	7	85	regulation of cytokine biosynthetic process
GO:0043412	0.00004	2.08616	25	45	2282	macromolecule modification
GO:0030217	0.00004	14.86076	0	5	39	T cell differentiation
GO:0070663	0.00004	5.92494	2	9	154	regulation of leukocyte proliferation
GO:0045621	0.00005	10.12288	1	6	62	positive regulation of lymphocyte differentiation
GO:0016265	0.00005	2.32191	15	32	1422	death
GO:0046640	0.00005	23.4375	0	4	20	regulation of alpha-beta T cell proliferation
GO:0050856	0.00005	23.4375	0	4	20	regulation of T cell receptor signaling pathway
GO:0043068	0.00007	3.21905	6	17	532	positive regulation of programmed cell death
GO:0032673	0.00007	22.05767	0	4	21	regulation of interleukin-4 production
GO:2000026	0.00007	2.47036	12	27	1117	regulation of multicellular organismal development
GO:0002460	0.00008	5.50396	2	9	165	adaptive immune response based on somatic recombination of immune receptors built from immunoglobulin superfamily domains
GO:0051716	0.00008	1.7509	57	83	5346	cellular response to stimulus
GO:0018108	0.00008	4.88173	2	10	206	peptidyl-tyrosine phosphorylation
GO:0032946	0.00009	7.37186	1	7	97	positive regulation of mononuclear cell proliferation
GO:0046631	0.00009	20.47476	0	4	23	alpha-beta T cell activation
GO:0006917	0.00009	4.07976	3	12	295	induction of apoptosis
GO:0042107	0.00009	7.29047	1	7	98	cytokine metabolic process
GO:0050857	0.0001	46.67073	0	3	9	positive regulation of antigen receptor-mediated signaling pathway

Supplemental Table 7.

Genes down-regulated in cycling T cells (day 4)

GOBPID	Pvalue	OddsRatio	ExpCount	Count	Size	Term
GO:0002684	0	9.33713	2	14	418	positive regulation of immune system process
GO:0050776	0	9.22173	2	12	354	regulation of immune response
GO:0019884	0	60.9019	0	5	25	antigen processing and presentation of exogenous antigen
GO:0019886	0	106.97778	0	4	13	antigen processing and presentation of exogenous peptide antigen via MHC class II
GO:0002252	0	8.51482	2	11	346	immune effector process
GO:0048002	0	40.58017	0	5	35	antigen processing and presentation of peptide antigen
GO:0042113	0	12.56908	1	8	168	B cell activation
GO:0002504	0	74.04615	0	4	17	antigen processing and presentation of peptide or polysaccharide antigen via MHC class II
GO:0006959	0	21.40022	0	6	75	humoral immune response
GO:0032943	0	11.09858	1	8	189	mononuclear cell proliferation
GO:0002757	0	13.07724	1	7	140	immune response-activating signal transduction
GO:0045089	0	16.95668	0	6	93	positive regulation of innate immune response
GO:0050670	0	12.33011	1	7	148	regulation of lymphocyte proliferation
GO:0070663	0	11.82313	1	7	154	regulation of leukocyte proliferation
GO:0045087	0.00001	11.03123	1	7	177	innate immune response
GO:0006950	0.00001	3.32145	9	23	1987	response to stress
GO:0050868	0.00001	19.6029	0	5	67	negative regulation of T cell activation
GO:0050867	0.00002	9.58513	1	7	188	positive regulation of cell activation
GO:0016064	0.00004	14.80627	0	5	87	immunoglobulin mediated immune response
GO:0002449	0.00005	10.07323	1	6	152	lymphocyte mediated immunity
GO:0051251	0.00007	9.42258	1	6	162	positive regulation of lymphocyte activation
GO:0002460	0.00008	9.24335	1	6	165	adaptive immune response based on somatic recombination of immune receptors built from immunoglobulin superfamily domains
GO:0002695	0.0001	12.00902	0	5	106	negative regulation of leukocyte activation

Supplemental Table 8.
Down regulated genes in cylcing B and T cells (day 4)

GOBPID	Pvalue	OddsRatio	ExpCount	Count	Size	Term
GO:0080090	0	2.22066	84	149	3828	regulation of primary metabolic process
GO:0051171	0	2.24085	67	123	3017	regulation of nitrogen compound metabolic process
GO:0051252	0	2.18627	56	104	2533	regulation of RNA metabolic process
GO:0035556	0	2.5116	32	71	1463	intracellular signal transduction
GO:0032774	0	2.02833	56	99	2550	RNA biosynthetic process
GO:0036211	0	2.09467	49	89	2202	protein modification process
GO:0048534	0	3.16594	12	35	554	hemopoietic or lymphoid organ development
GO:0001775	0	3.01635	13	35	579	cell activation
GO:0010627	0	3.04833	12	33	539	regulation of intracellular protein kinase cascade
GO:0018193	0	2.8953	13	34	583	peptidyl-amino acid modification
GO:0009966	0	2.39564	21	46	1009	regulation of signal transduction
GO:0034645	0	1.77418	72	112	3271	cellular macromolecule biosynthetic process
GO:0016310	0	2.09951	31	60	1427	phosphorylation
GO:0010556	0	2.06894	34	63	1622	regulation of macromolecule biosynthetic process
GO:0031326	0	2.03705	34	63	1650	regulation of cellular biosynthetic process
GO:0002684	0	3.06491	9	26	418	positive regulation of immune system process
GO:0002694	0	3.5598	6	21	292	regulation of leukocyte activation
GO:0048523	0	1.77365	58	92	2629	negative regulation of cellular process
GO:0044093	0	2.2335	22	45	993	positive regulation of molecular function
GO:0006357	0.00001	2.10206	26	50	1173	regulation of transcription from RNA polymerase II promoter
GO:0043065	0.00001	2.69569	12	29	527	positive regulation of apoptotic process
GO:0090304	0.00001	1.65593	79	115	3560	nucleic acid metabolic process
GO:0050790	0.00001	1.97792	32	58	1451	regulation of catalytic activity
GO:0034641	0.00001	1.59581	102	141	4607	cellular nitrogen compound metabolic process
GO:0031329	0.00001	2.75824	11	27	479	regulation of cellular catabolic process
GO:0046649	0.00001	2.85054	9	25	429	lymphocyte activation
GO:0010467	0.00001	1.61989	83	119	3763	gene expression
GO:0006955	0.00001	2.49783	13	31	606	immune response
GO:0010942	0.00001	2.58366	12	29	548	positive regulation of cell death
GO:0043123	0.00002	5.41205	2	11	103	positive regulation of I-kappaB kinase/NF-kappaB cascade
GO:0009893	0.00002	1.83199	40	67	1812	positive regulation of metabolic process
GO:0009058	0.00002	1.58493	92	128	4152	biosynthetic process
GO:0001932	0.00002	2.3387	16	34	709	regulation of protein phosphorylation
GO:0051270	0.00002	2.77891	9	24	421	regulation of cellular component movement
GO:0044267	0.00002	1.66711	63	94	2834	cellular protein metabolic process
GO:0050793	0.00002	1.92292	31	55	1406	regulation of developmental process
GO:0043547	0.00003	3.47679	5	17	240	positive regulation of GTPase activity
GO:0050789	0.00003	1.53812	148	187	7155	regulation of biological process
GO:0006355	0.00003	1.99843	27	49	1287	regulation of transcription, DNA-dependent
GO:0046578	0.00003	3.31387	6	18	266	regulation of Ras protein signal transduction
GO:0030099	0.00003	3.43009	5	17	243	myeloid cell differentiation
GO:0043067	0.00003	2.01835	25	46	1114	regulation of programmed cell death
GO:0018107	0.00003	7.35395	1	8	57	peptidyl-threonine phosphorylation
GO:0008360	0.00004	5.50974	2	10	92	regulation of cell shape
GO:0031328	0.00004	1.96503	27	49	1220	positive regulation of cellular biosynthetic process
GO:0050863	0.00004	3.92551	4	14	176	regulation of T cell activation
GO:0002274	0.00004	4.92743	2	11	112	myeloid leukocyte activation
GO:0010557	0.00004	1.99068	25	46	1128	positive regulation of macromolecule biosynthetic process
GO:0006911	0.00005	16.00203	0	5	19	phagocytosis, engulfment
GO:0033124	0.00005	3.15889	6	18	278	regulation of GTP catabolic process
GO:0002521	0.00006	2.9121	7	20	334	leukocyte differentiation
GO:0006793	0.00006	1.78879	38	62	1703	phosphorus metabolic process
GO:0019220	0.00007	2.11592	19	37	849	regulation of phosphate metabolic process
GO:0010035	0.00007	3.07492	6	18	285	response to inorganic substance
GO:0048584	0.00007	2.03192	22	41	981	positive regulation of response to stimulus
GO:0001817	0.00007	2.86568	7	20	339	regulation of cytokine production
GO:0002697	0.00007	3.67375	4	14	187	regulation of immune effector process
GO:0045619	0.00009	4.90825	2	10	102	regulation of lymphocyte differentiation
GO:0051247	0.00009	2.21722	15	32	699	positive regulation of protein metabolic process
GO:0051592	0.00009	6.31914	1	8	65	response to calcium ion

Supplemental Table 9.

Upregulated genes in B cells (resting and cycling cells)

GOBPID	Pvalue	OddsRatio	ExpCount	Count	Size	Term
GO:0006955	0	5.54409	10	47	606	immune response
GO:0002764	0	10.73164	3	22	149	immune response-regulating signaling pathway
GO:0050853	0	36.75176	0	11	29	B cell receptor signaling pathway
GO:0002253	0	7.67997	3	20	180	activation of immune response
GO:0002504	0	52.97945	0	8	17	antigen processing and presentation of peptide or polysaccharide antigen via MHC class II
GO:0002252	0	6.09514	4	21	238	immune effector process
GO:0006793	0	2.52925	29	63	1703	phosphorus metabolic process
GO:0016310	0	2.60126	24	55	1427	phosphorylation
GO:0042113	0	10.75273	1	12	82	B cell activation
GO:0002429	0	10.02646	1	12	84	immune response-activating cell surface receptor signaling pathway
GO:0002460	0	6.5165	3	16	165	adaptive immune response based on somatic recombination of immune receptors built from immunoglobulin superfamily domains
GO:0050670	0	6.82801	3	15	148	regulation of lymphocyte proliferation
GO:0019886	0	50.77437	0	6	13	antigen processing and presentation of exogenous peptide antigen via MHC class II
GO:0070663	0	6.5312	3	15	154	regulation of leukocyte proliferation
GO:0009617	0	4.35393	6	22	332	response to bacterium
GO:0065009	0	2.24815	31	62	1855	regulation of molecular function
GO:0001816	0	3.93316	6	23	382	cytokine production
GO:0002684	0	4.3122	5	20	310	positive regulation of immune system process
GO:0051704	0	2.96015	12	31	681	multi-organism process
GO:0002694	0	4.23661	5	19	292	regulation of leukocyte activation
GO:0050864	0	9.90706	1	9	64	regulation of B cell activation
GO:0048002	0	14.83906	1	7	35	antigen processing and presentation of peptide antigen
GO:0048584	0	2.52086	17	38	981	positive regulation of response to stimulus
GO:0002449	0	5.62432	3	13	152	lymphocyte mediated immunity
GO:0002520	0	3.00022	10	27	581	immune system development
GO:0002281	0	32.80366	0	5	14	macrophage activation involved in immune response
GO:0030889	0	32.80366	0	5	14	negative regulation of B cell proliferation
GO:0032496	0	5.0197	3	14	182	response to lipopolysaccharide
GO:0019884	0	18.69454	0	6	25	antigen processing and presentation of exogenous antigen
GO:0035556	0	2.19489	25	49	1463	intracellular signal transduction
GO:0043547	0	4.31741	4	16	240	positive regulation of GTPase activity
GO:0030811	0	3.84343	5	18	302	regulation of nucleotide catabolic process
GO:0006954	0	3.52679	6	20	365	inflammatory response
GO:0010604	0.00001	2.07783	28	53	1673	positive regulation of macromolecule metabolic process
GO:0071900	0.00001	3.78024	5	17	289	regulation of protein serine/threonine kinase activity
GO:0002758	0.00001	8.80901	1	8	62	innate immune response-activating signal transduction
GO:0034110	0.00001	22.70545	0	5	18	regulation of homotypic cell-cell adhesion
GO:0043549	0.00001	2.92217	9	24	526	regulation of kinase activity
GO:0002366	0.00001	5.48318	2	11	131	leukocyte activation involved in immune response
GO:0065008	0.00001	1.91848	35	61	2092	regulation of biological quality
GO:0002224	0.00001	10.12706	1	7	48	toll-like receptor signaling pathway
GO:0009607	0.00002	3.3421	6	19	374	response to biotic stimulus
GO:0071216	0.00002	5.43757	2	11	132	cellular response to biotic stimulus
GO:0016064	0.00002	6.87373	1	9	87	immunoglobulin mediated immune response
GO:0010543	0.00002	19.67598	0	5	20	regulation of platelet activation
GO:0036211	0.00002	1.88354	37	63	2202	protein modification process
GO:0009894	0.00002	2.82386	9	24	543	regulation of catabolic process
GO:0043085	0.00002	3.06905	7	21	455	positive regulation of catalytic activity
GO:0051347	0.00002	3.3937	6	18	339	positive regulation of transferase activity
GO:0033124	0.00002	3.68376	5	16	278	regulation of GTP catabolic process
GO:0031663	0.00002	12.2417	1	6	35	lipopolysaccharide-mediated signaling pathway
GO:0006952	0.00002	3.34908	6	18	359	defense response
GO:0010033	0.00002	2.07564	24	45	1403	response to organic substance
GO:0045577	0.00003	17.35934	0	5	22	regulation of B cell differentiation
GO:0045089	0.00003	6.38073	2	9	93	positive regulation of innate immune response
GO:0051241	0.00003	3.44564	5	17	315	negative regulation of multicellular organismal process
GO:0030890	0.00003	11.83302	1	6	36	positive regulation of B cell proliferation
GO:0031323	0.00003	1.68074	65	96	3867	regulation of cellular metabolic process
GO:0043406	0.00003	4.98154	2	11	143	positive regulation of MAP kinase activity
GO:0044270	0.00003	2.46658	13	29	751	cellular nitrogen compound catabolic process
GO:0032655	0.00003	11.45071	1	6	37	regulation of interleukin-12 production
GO:0032946	0.00004	6.08941	2	9	97	positive regulation of mononuclear cell proliferation
GO:0030098	0.00004	3.89427	4	14	230	lymphocyte differentiation
GO:0006470	0.00005	4.76344	3	11	149	protein dephosphorylation
GO:0019220	0.00005	2.33068	14	31	849	regulation of phosphate metabolic process
GO:0045576	0.00005	10.4387	1	6	40	mast cell activation
GO:0080090	0.00006	1.65177	65	94	3828	regulation of primary metabolic process
GO:0045860	0.00007	3.34655	5	16	304	positive regulation of protein kinase activity
GO:0001782	0.00007	14.04984	0	5	26	B cell homeostasis
GO:0046700	0.00007	2.39772	13	28	743	heterocycle catabolic process
GO:0045321	0.00007	4.53924	3	11	166	leukocyte activation
GO:0030097	0.00007	2.69974	9	22	517	hemopoiesis
GO:0001932	0.00008	2.42023	12	27	709	regulation of protein phosphorylation
GO:0023051	0.00009	1.87098	30	52	1798	regulation of signaling
GO:0012501	0.00009	2.00401	23	42	1345	programmed cell death
GO:0050867	0.00009	4.07922	3	12	188	positive regulation of cell activation
GO:1901292	0.00009	2.39834	12	27	715	nucleoside phosphate catabolic process
GO:0002700	0.00009	7.40859	1	7	63	regulation of production of molecular mediator of immune response
GO:0002699	0.00009	5.40966	2	9	108	positive regulation of immune effector process
GO:0032844	0.0001	3.56046	4	14	250	regulation of homeostatic process
GO:0042454	0.0001	2.87925	7	19	418	ribonucleoside catabolic process

Supplemental table 10

Downregulated genes in T cells (Day 0)

GOBPID	Pvalue	OddsRatio	ExpCount	Count	Size	Term
GO:0006281	0,00003	6,35353	2	9	321	DNA repair
GO:0008152	0,00003	2,39456	45	65	9211	metabolic process
GO:0044257	0,00004	6,15297	2	9	331	cellular protein catabolic process
GO:0006511	0,00008	6,34617	1	8	283	ubiquitin-dependent protein catabolic process
GO:0043632	0,0001	6,14214	1	8	292	modification-dependent macromolecule catabolic process

Supplemental Table 11.

Upregulated genes in cycling B cells (day 4)

GOBPID	Pvalue	OddsRatio	ExpCount	Count	Size	Term
GO:0002376	0.00001	2.9463	10	26	1264	immune system process

Supplemental table 12. 4C sequencing primers

Supplemental table 12a. 4C single-end sequencing primers

4C viewpoint	Primer type	Primer sequence
Upstream IgH	Primer 1	AATGATACGGCGACCACCGAACACTCTTTCCCTACACGACGCTCTTCCGATCTTCAAGGCTAATGAAGCTT
Upstream IgH	Primer 2	CAAGCAGAAGACGGCATACGATCAGCGGGGATGTAGAGC

Supplemental table 12b. 4C paired-end sequencing primers

4C viewpoint	Primer type	Primer sequence
3'RR_4C capture	PE 1	AATGATACGGCGACCACCGAGATCTACACTCTTTCCCTACACGACGCTCTTCCGATCTCCGAGGCCTTACAAGCTT
3'RR_SNP	PE 2	CAAGCAGAAGACGGCATACGAGATCGGTCTCGGCATTCCTGCTGAACCGCTCTTCCGATCTCTAACTTTGGCCCAGAAATC
IgG3_4C capture	PE 1	AATGATACGGCGACCACCGAGATCTACACTCTTTCCCTACACGACGCTCTTCCGATCTAAGCCTTTCTAAGGCAGATC
IgG3_SNP	PE 2	CAAGCAGAAGACGGCATACGAGATCGGTCTCGGCATTCCTGCTGAACCGCTCTTCCGATCTGGGACATTAGAGACATAGCTG
Distal V_4C capture	PE 1	AATGATACGGCGACCACCGAGATCTACACTCTTTCCCTACACGACGCTCTTCCGATCTGGTGATTTGCATTTCATAGATC
Distal V_SNP	PE 2	CAAGCAGAAGACGGCATACGAGATCGGTCTCGGCATTCCTGCTGAACCGCTCTTCCGATCTCGGGCCTCATATCAGCTA

Supplemental table 13. Chromosomal locations of the SNPs (mm9)

4C viewpoint name	Chr	Position	C57Bl/6 sequence	FVB sequence
3RR	12	114495014	T	C
Cγ3	12	114594500	C	T
Distal V	12	119923316	A	G

Supplemental figure 1.

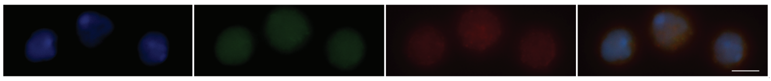

DAPI, IgH, CD45, overlay

Supplemental figure 2.

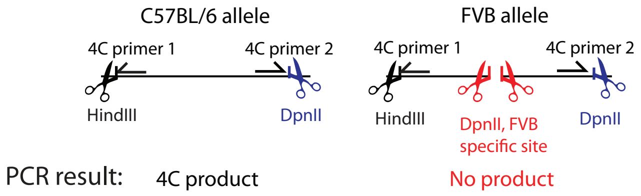

Supplemental figure 3

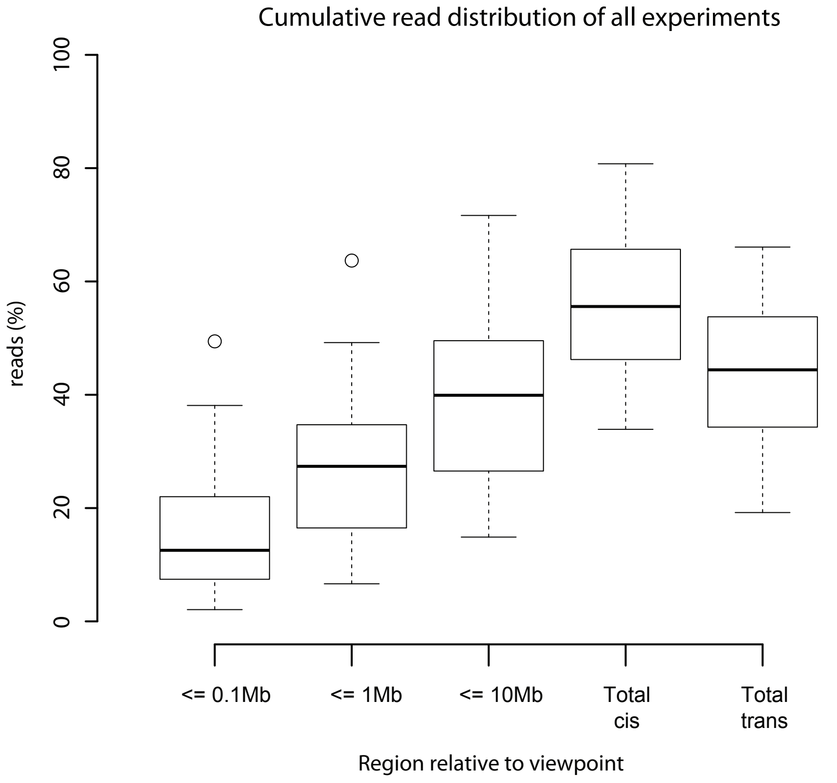

Supplemental figure 4

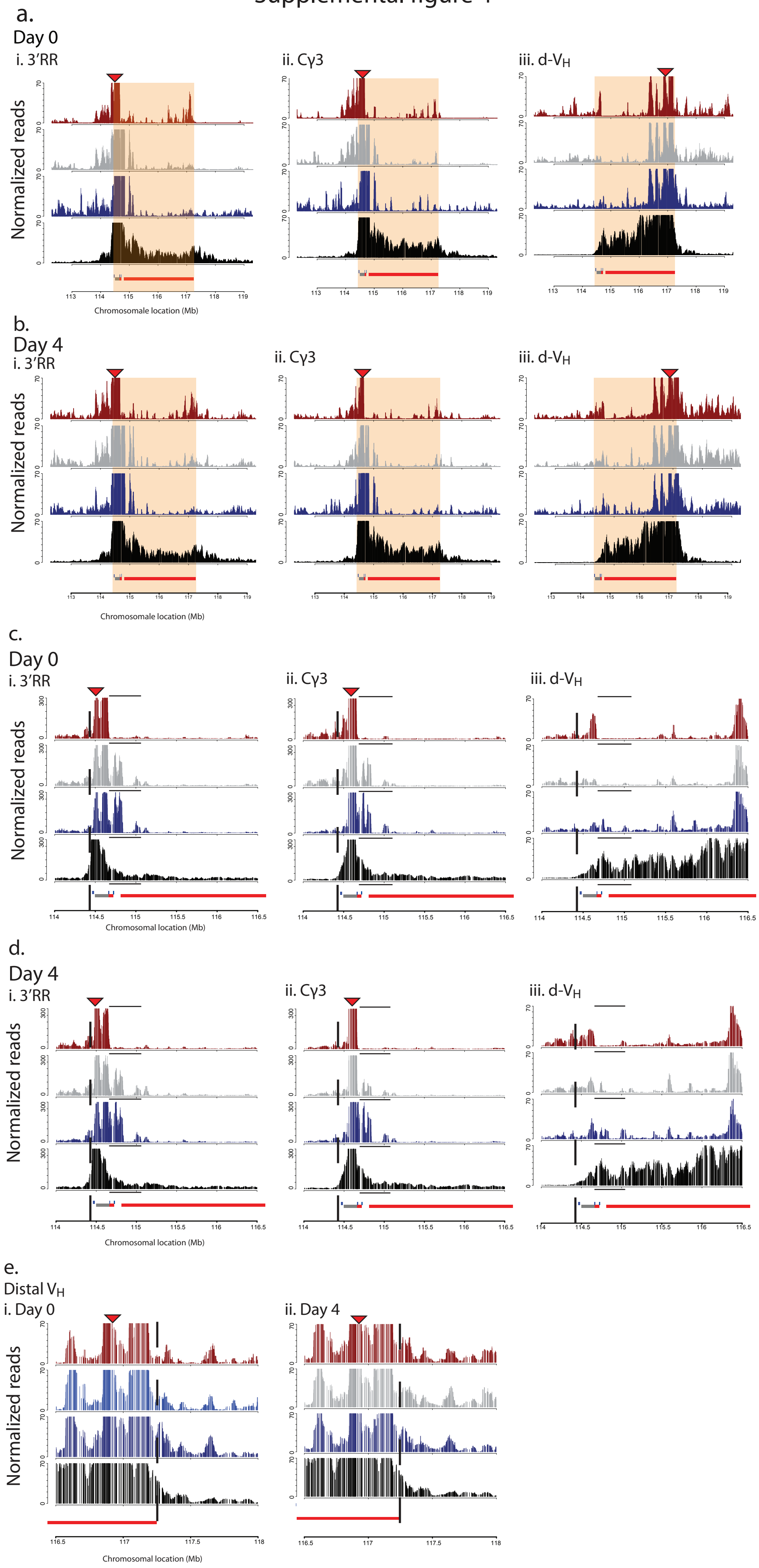

Supplemental figure 5.

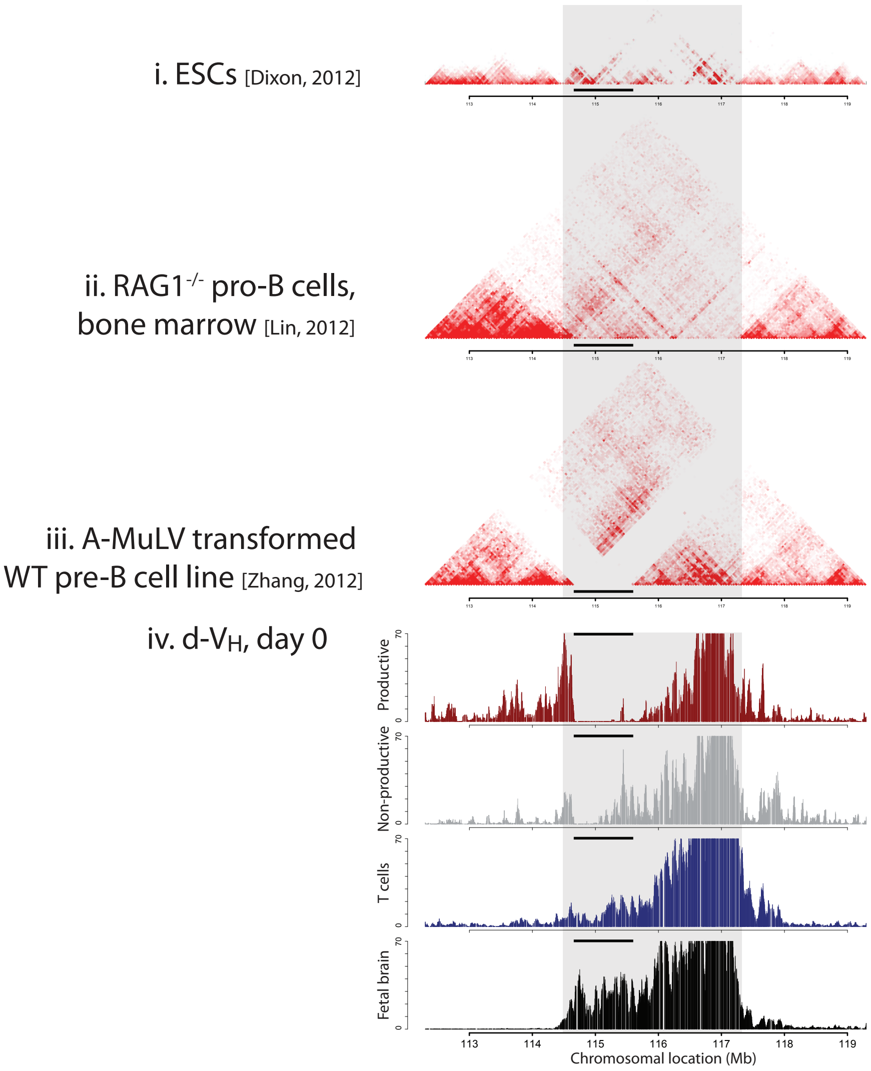

Supplemental Figure 6.

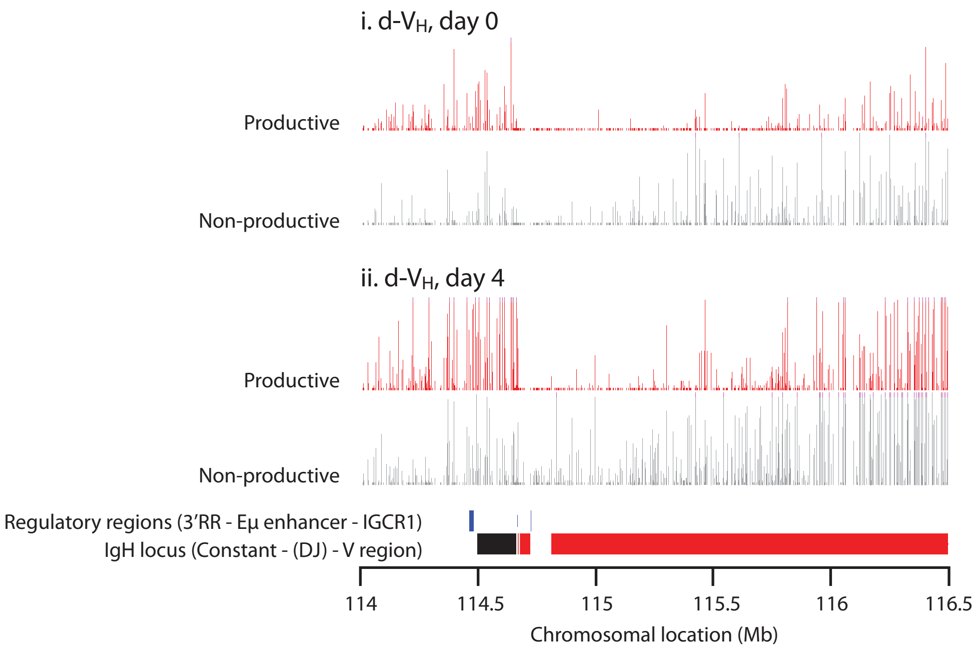

Supplemental figure 7

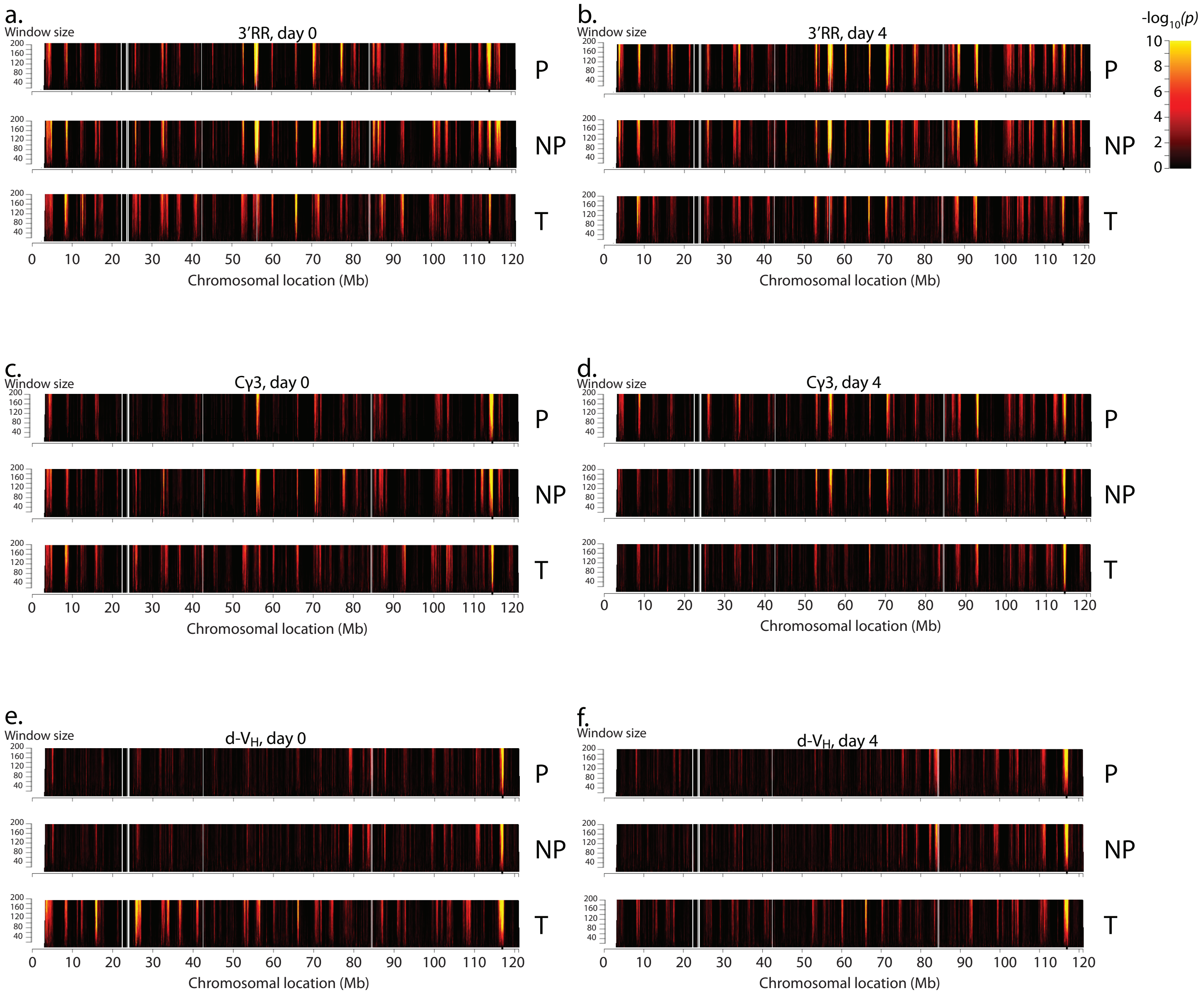

Supplemental figure 8.

a.

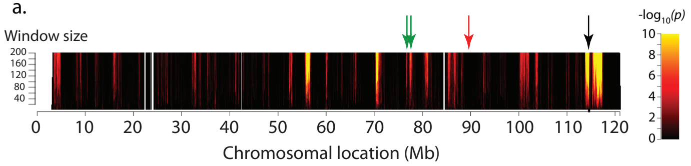

interaction A: chr12 77.5 - 77.7 Mb
interaction B: chr12 78.9 - 79.1 Mb
negative control: chr12 89.6 - 89.8 Mb
IgH locus: chr12 114.5 - 114.7 Mb

b.

i. interaction A (DAPI, A, IgH)

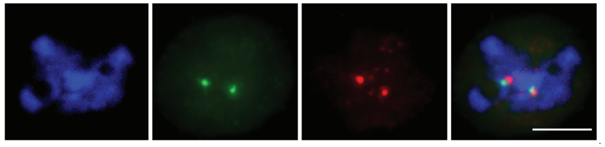

ii. negative control (DAPI, neg ctrl., IgH)

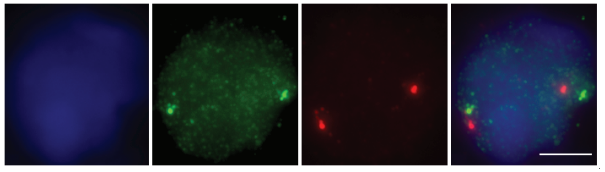

c.

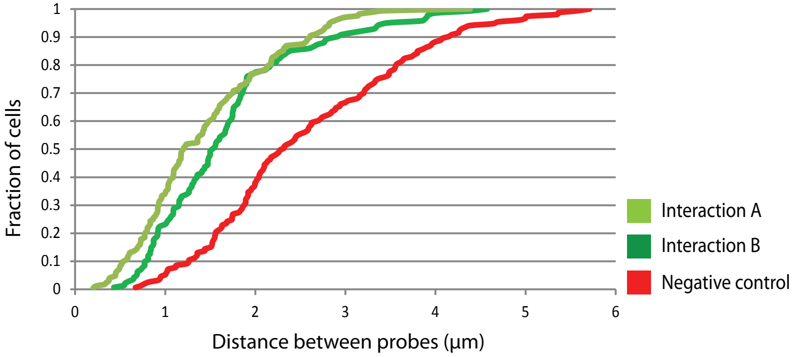

Supplemental figure 9

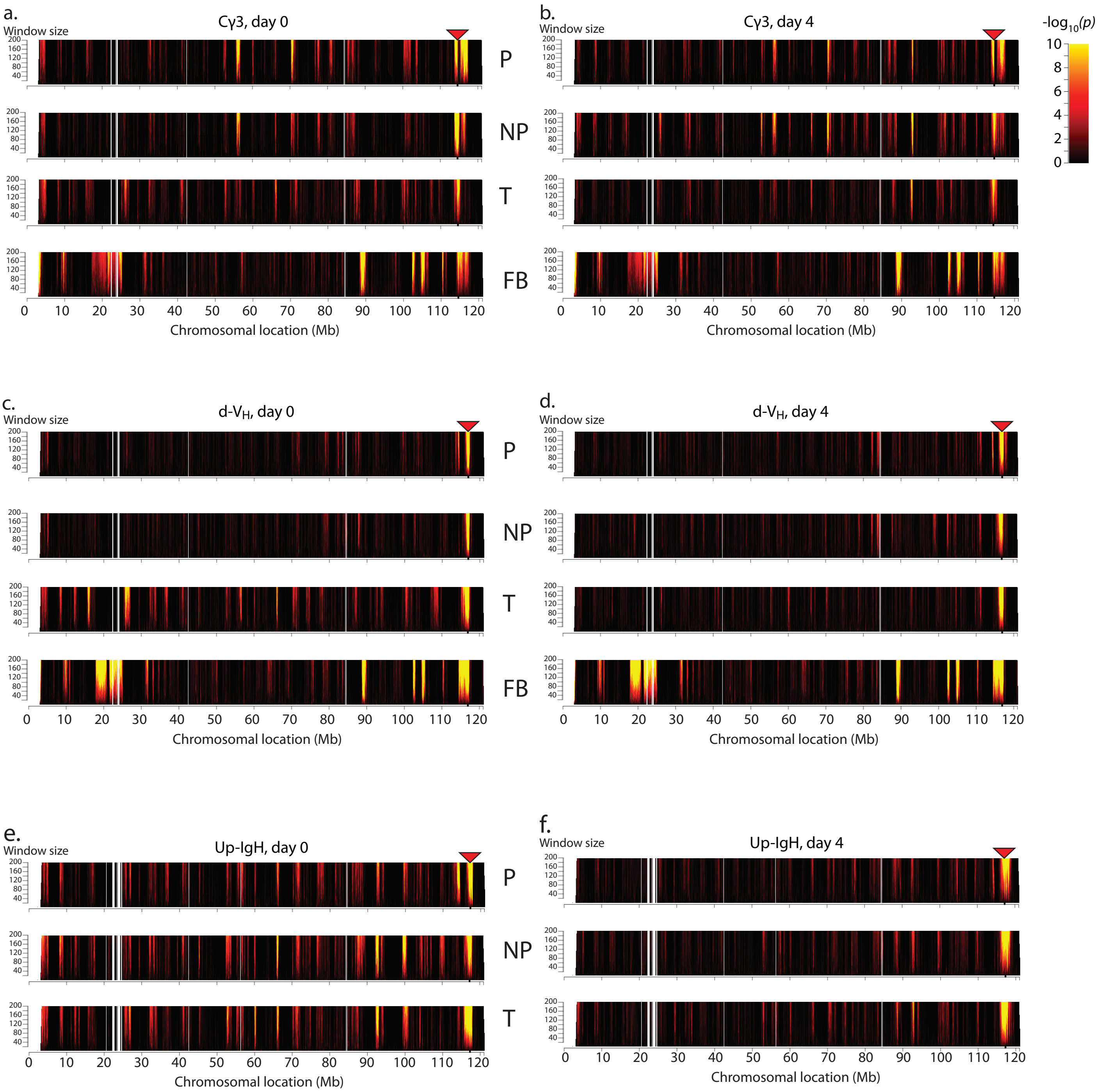

Supplemental figure 10

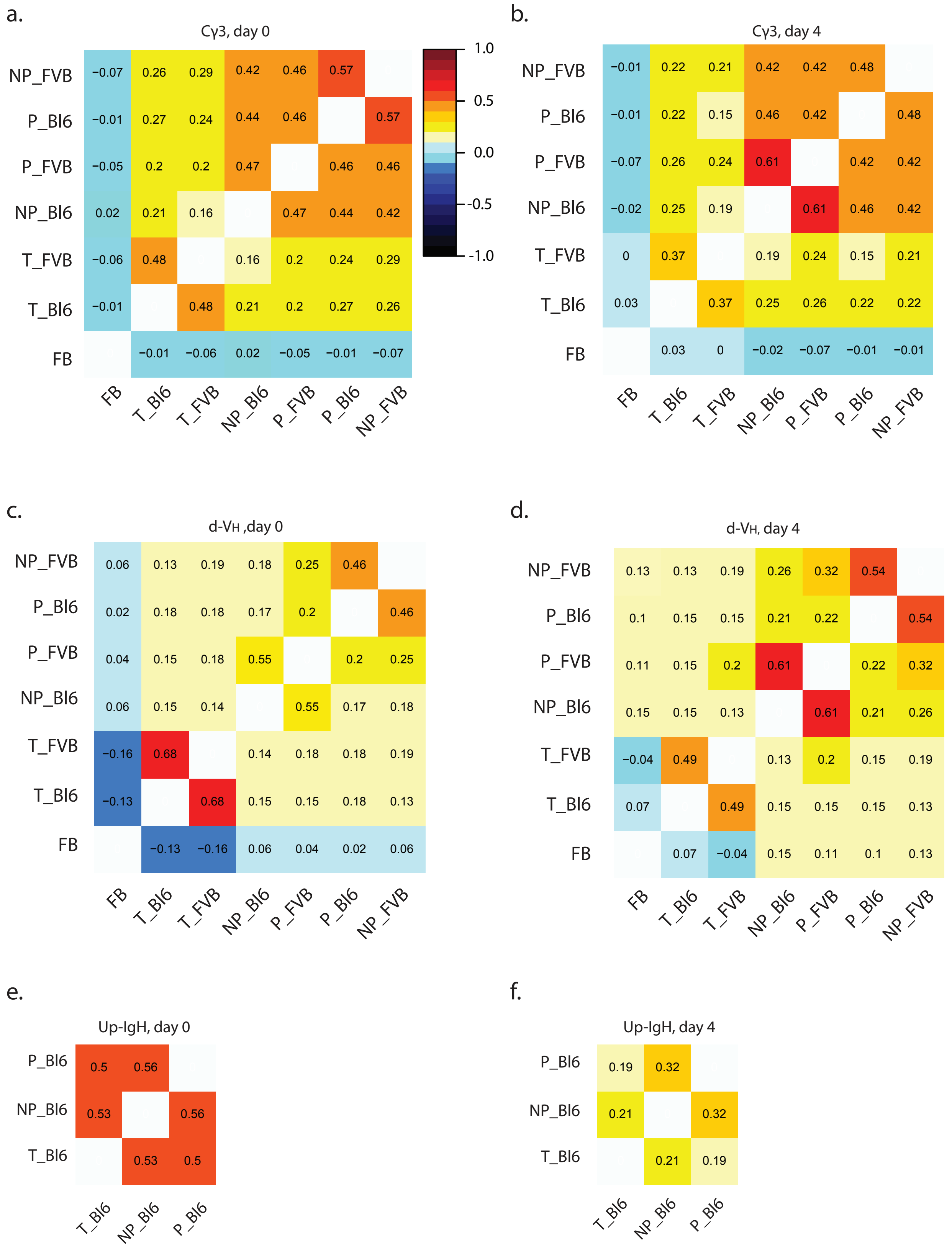

Supplemental figure 11.

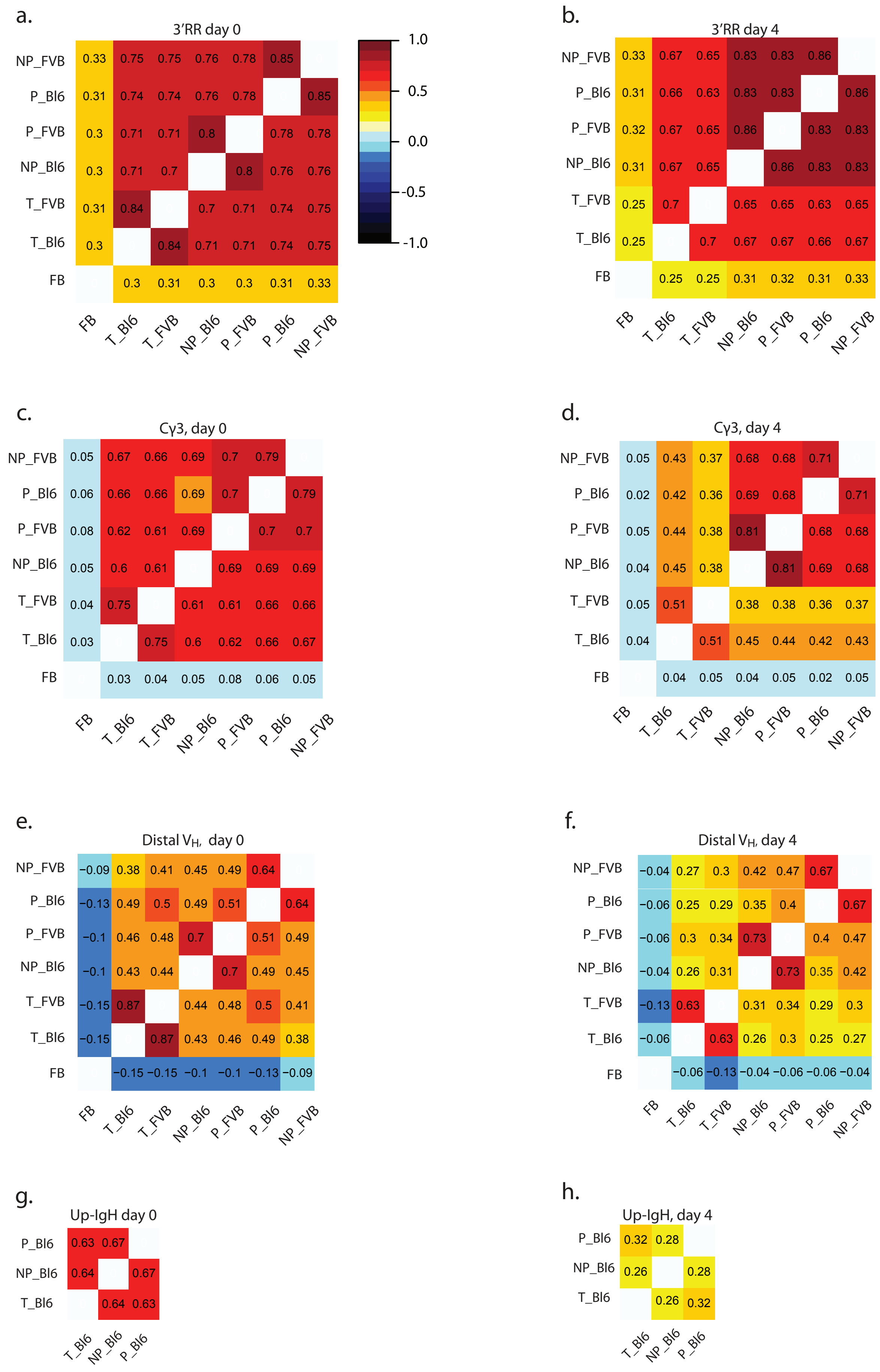

Supplemental Figure 12.

a.

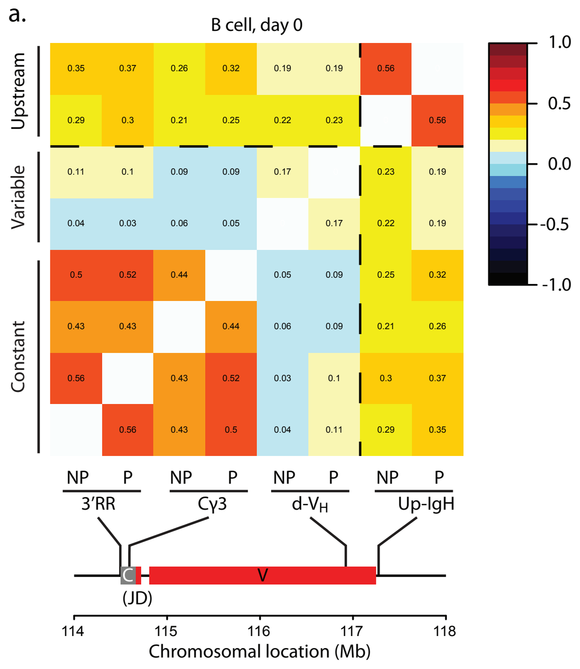

b.

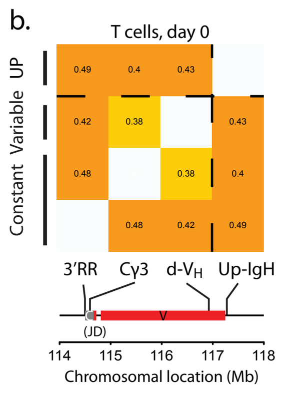

Supplemental Figure 13.

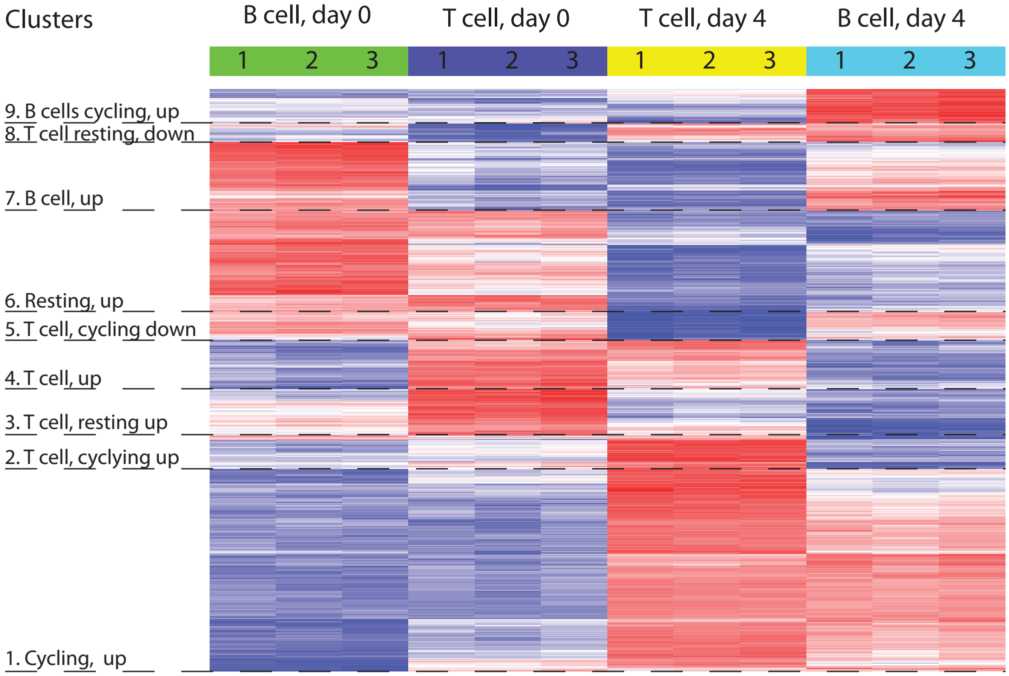

Supplement: Supplementary Data [file supp_gkt491_nar-00999-m-2013-File008.pdf]
